# Proteogenomic Workflow Reveals Molecular Phenotypes Related to Breast Cancer Mammographic Appearance

**DOI:** 10.1021/acs.jproteome.1c00243

**Published:** 2021-04-15

**Authors:** Tommaso De Marchi, Paul Theodor Pyl, Martin Sjöström, Stina Klasson, Hanna Sartor, Lena Tran, Gyula Pekar, Johan Malmström, Lars Malmström, Emma Niméus

**Affiliations:** † Division of Surgery, Oncology, and Pathology, Department of Clinical Sciences, Lund University, Solvegatan 19, Lund SE-223 62, Sweden; ‡ Department Plastic and Reconstructive Surgery, Skåne University Hospital, Inga Marie Nilssons gata 47, Malmö SE-20502, Sweden; § Division of Diagnostic Radiology, Department of Translational Medicine, Skåne University Hospital, Entrégatan 7, Lund SE-22185, Sweden; ∥ Division of Oncology and Pathology, Department of Clinical Sciences, Lund University, Skåne University Hospital, Lund SE-22185, Sweden; ⊥ Division of Infection Medicine, Department of Clinical Sciences Lund, Faculty of Medicine, Lund University, Klinikgatan 32, Lund SE-22184, Sweden; # S3IT, University of Zurich, Winterthurerstrasse 190, Zurich CH-8057, Switzerland; ∇ Institute for Computational Science, University of Zurich, Winterthurerstrasse 190, Zurich CH-8057, Switzerland; ○ Department of Surgery, Skåne University Hospital, Lund 222 42, Sweden

**Keywords:** data-independent acquisition, proteogenomics, breast cancer, transcriptomics, proteomics

## Abstract

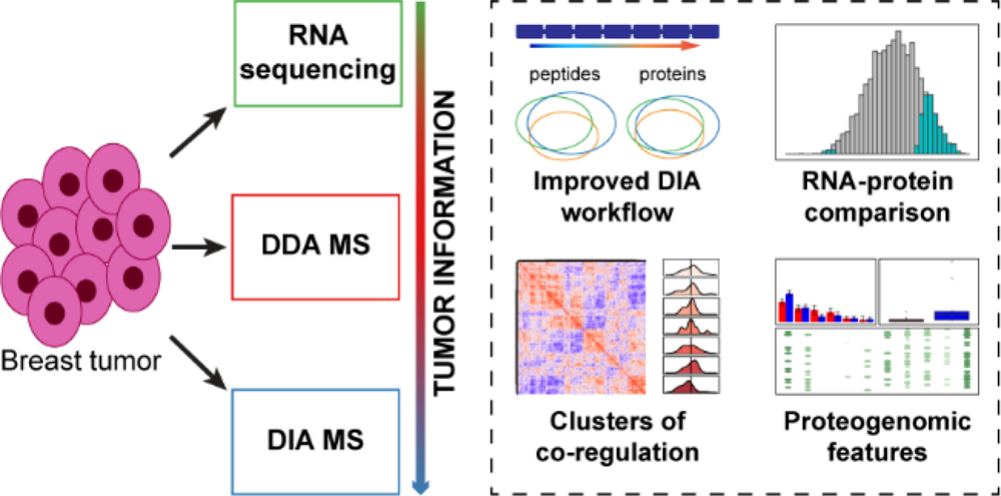

Proteogenomic approaches have enabled the generat̲ion of novel information levels when compared to single omics studies although burdened by extensive experimental efforts. Here, we improved a data-independent acquisition mass spectrometry proteogenomic workflow to reveal distinct molecular features related to mammographic appearances in breast cancer. Our results reveal splicing processes detectable at the protein level and highlight quantitation and pathway complementarity between RNA and protein data. Furthermore, we confirm previously detected enrichments of molecular pathways associated with estrogen receptor-dependent activity and provide novel evidence of epithelial-to-mesenchymal activity in mammography-detected spiculated tumors. Several transcript–protein pairs displayed radically different abundances depending on the overall clinical properties of the tumor. These results demonstrate that there are differentially regulated protein networks in clinically relevant tumor subgroups, which in turn alter both cancer biology and the abundance of biomarker candidates and drug targets.

## Introduction

Breast cancer (BC) is the most common female malignancy. BC is associated with increasing incidence rates, but the mortality is steadily decreasing due to better patient care, the availability of new treatment options, and a deeper understanding of the mutational and molecular dynamics of each breast cancer type.^1,2^ BCs are broadly classified according to the status of the estrogen and progesterone receptors (ER and PgR), the receptor tyrosine kinase ERBB2 and Ki67. More recent works based on transcriptome analysis have enabled the definition of intrinsic (luminal A, luminal B, normal-like, Her2, and basal)^3^ and molecular driver-related^4^ subtypes, which are used to predict patient prognosis and to guide treatment.^5^

Mammographic imaging is a diagnostic modality used for early tumor detection. Improved mammographic image analysis has furthermore revealed that breast cancers can manifest different appearances, such as spiculations. Spiculated BCs have a star-like appearance, which is an indicator for invasiveness, cancer infiltration, and fibrotic growth around the tumor.^6,7^ Typically, spiculated tumors are overrepresented in the ER+/PgR+ and luminal A tumor group and have been linked to better prognosis when compared to well-defined and microcalcified masses.^8−10^ These findings indicate that there are both receptor status- and intrinsic subtype-dependent molecular drivers that contribute to spiculated appearances. However, the relationship between mutational events and downstream protein regulation patterns responsible for spiculation has remained uncharacterized.

Previous breast cancer reports have shown that the integration of genomic/transcriptomic and proteomic data, referred to as proteogenomics, can play an important role in the definition of new molecular drivers in breast cancer. For example, previous studies have identified protein-level evidence of genomic aberrations such as chromosomal losses, defined new BC subgroups such as G-protein-coupled receptors, identified new antigens for immunotherapy, and investigated abundance discrepancies between transcript and protein pairs in molecular pathways (e.g., metabolism and coagulation).^11−13^ Interestingly, several of these studies showed that there are marked discrepancies in transcript and protein abundances that relate to particular molecular tumor subtypes and protein classes. In contrast, tumor subgroup-dependent features and their influence on RNA and protein abundance have been sparsely investigated, with little focus of their impact on biomarker measurement, drug target monitoring, or immunotherapy epitope expression.

So far, most proteogenomic studies in BC have relied on peptide fractionation followed by extensive data-dependent acquisition (DDA) MS analysis, typically associated with high instrument usage. However, a recent study employed data-independent acquisition (DIA) MS to define high-quality protein maps of BC subtypes. The study by Bouchal *et al.* identified the protein markers INPP4B, CDK1, and ERBB2 as discriminatory of key BC histopathological features (e.g., lymph-node status), pinpointed pathways that relates to tumor phenotypes, and assessed the degree of similarity between transcript and protein abundance.^14^ In DIA MS (or sequential window acquisition of all theoretical mass spectra (SWATH)^15,16^), consistent peptide/protein identification rates is achieved across samples via DDA-based spectral libraries.

The continuously increasing coverage of the breast cancer proteome achieved by the research community provides new opportunities to increase and refine breast cancer-specific spectral libraries to further improve DIA-based quantification. In addition, the use of spectral libraries has been shown to reduce false discovery rates (FDR) for the identification of proteins and their isoforms or mutation-defined single amino acid variants (SAAVs) by searching against a smaller database of previously observed peptides rather than relying on the whole-proteome search space.^17^

Here, we improved a previously established DIA MS-based proteogenomic workflow and used the workflow on a breast cancer cohort to identify molecular pathways related to breast cancer biology and mammographic appearances. The improved DIA MS-based workflow in combination with RNA-seq data from the same primary breast cancer tissues revealed novel molecular driver candidates of relevance for the receptor status and morphological appearance in breast cancer. The most notable feature was related to the enrichment of the epithelial-to-mesenchymal transition (EMT) pathway in spiculated tumors. These findings are in line with the concept that protrusions, i.e., spiculae form the invading front of the cancer, can remodel the surrounding healthy breast tissue.

## Experimental Procedures

### Experimental Design

**Figure 1:**
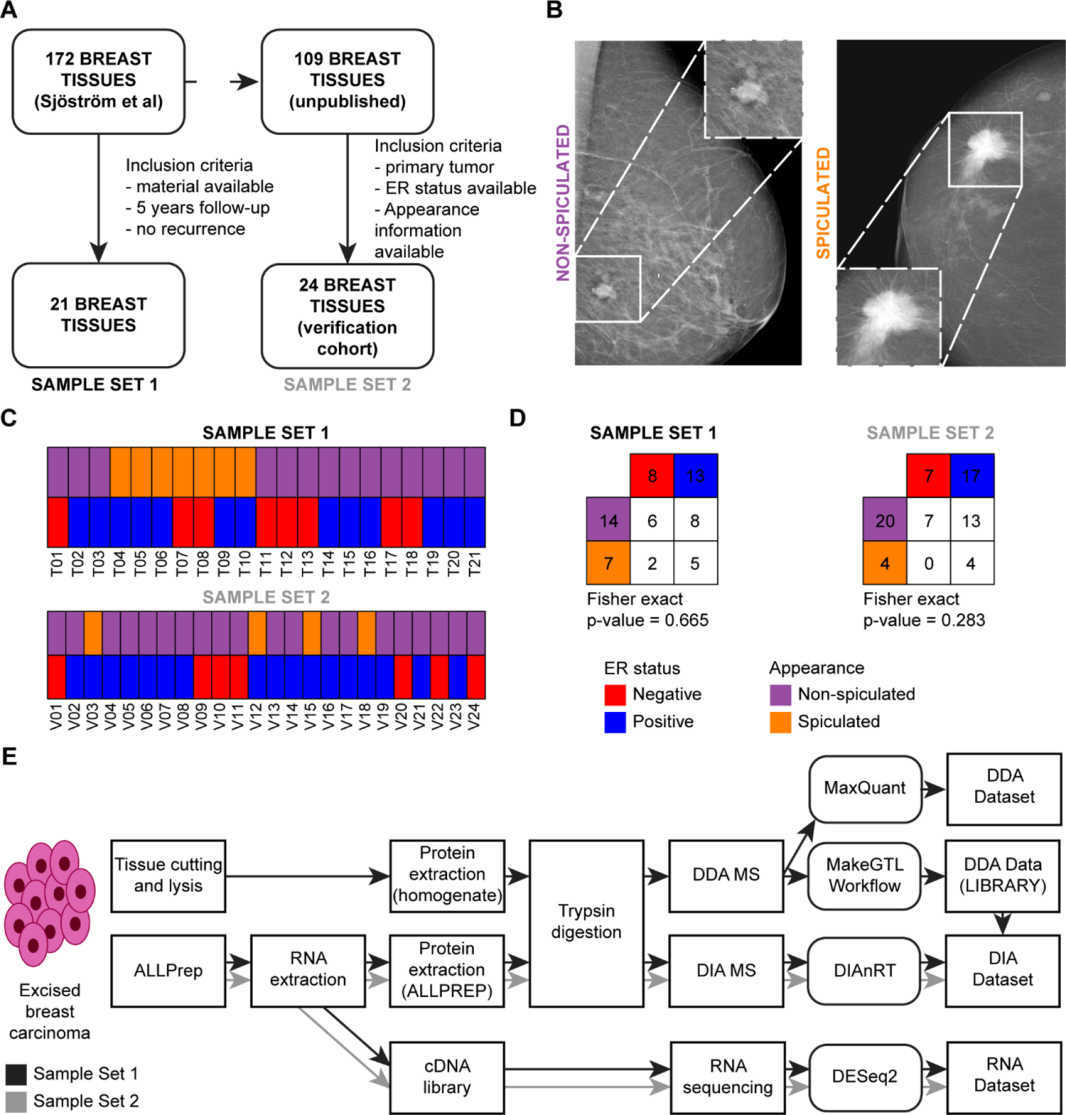
Experimental workflow of this study. A total of 21 samples derived from a larger cohort (set 1, *N* = 172, see Experimental Procedures) and a second set of 24 tumors from a larger study (set 2, *N* = 109, see Experimental Procedures) were employed (A). Panel (B) shows examples of nonspiculated and spiculated tumor masses. Panel (C) displays the overlap between the molecular (ER status) and appearance features evaluated in this study, for which no association was found (set 1: Fisher exact *p*-value = 0.665, set 2: Fisher exact *p*-value = 0.283, (D)). Tumor specimens were processed as whole tissue lysates (WTL, MS-only analysis) and ALLPREP flow-throughs (FT, RNA-seq and MS analyses). Panel (E) displays the experimental workflow of our RNA and MS (DDA and DIA) analyses: tumor tissues were cut into slices and processed by ALLPREP. RNA and protein fractions were extracted and processed from ALLPREP sample preparation for downstream RNA-sequencing and DDA/DIA MS, respectively. Tissue slices were prepared only for downstream MS (DDA/DIA). Samples for DDA were fractionated using strong anion exchange columns (SAX, six fractions) to enable higher proteome coverage. DDA data (i) was submitted to with MaxQuant processing to derive protein abundances and (ii) to the MakeGTL workflow to generate a spectral library for downstream DIA search. RNA-seq data was processed using the standard DESeq2 workflow (see Experimental Procedures). Abbreviations: DDA: data-dependent acquisition, DIA: data-independent acquisition, ER: estrogen receptor, FDR: false discovery rate, FT: flow-through, MS: mass spectrometry, RT: retention time, WTL: whole tissue lysate.

Two samples sets of breast cancer tissues (set 1: 21 samples and set 2: 24 samples) were analyzed by RNA-sequencing and MS analyses. DDA MS analysis was performed for downstream spectral library generation for downstream DIA MS (Figure 1). Samples were processed as ALLPREP flow-throughs (FT) and whole tissue lysates (WTL). FTs were analyzed by RNA-sequencing, DDA MS, and DDA MS. WTL samples were analyzed using DDA MS and merged with their FT counterparts to maximize the number of peptides included in the spectral library. Two external datasets were also employed (Tyanova^13^ and Bouchal^14^ datasets) were employed to maximize the search space for the DIA MS data. The RNA, DDA, and DIA datasets constituted the basis for all the analyses described in this manuscript.

### Patients

Out of a large breast cancer dataset consisting of 172 samples, a subset of 21 frozen breast tissue specimens was collected for this study (Figure 1A).^18^ All specimens were collected from women with primary breast cancer, who underwent tumor resection between 1991 and 2004. Estrogen and progesterone receptor (ER and PgR) statuses were assessed in tumor tissues by quantitative biochemical assays. The tumor content was derived from microscopic analysis of the tumor cell area in hematoxylin–eosin-stained tissue slices by two independent trained researchers. Breast cancer molecular subtype classification was derived from previously acquired expression data^18^ using the AIMS^19^ algorithm. IntClust^4^ classification was derived by processing the RNA-seq data using the IC10 package (v1.5).

The most dominant mammographic appearance of the tumor was retrospectively collected by one specialist in radiology (HS). Tumors were categorized as spiculated or other tumor appearances such as microcalcifications or well-defined masses (nonspiculated) based on their most dominating mammographic feature (i.e., appearance categorization). Examples of digital mammographic images are shown in Figure 1B. An overlap between spiculation and ER statuses is displayed in Figure 1C,D. A set of four normal breast tissues was also collected after a breast-reduction surgery at Lund University Hospital and used to generate the DIA spectral library. This subset of samples was selected based on the following clinical criteria: a breast volume of more than 800 mL, no previous case nor familial history of breast or ovarian cancer, nonsmoker, no diabetes mellitus, and a body mass index below 30.

An additional dataset of 24 primary breast tumors was derived from a separate study (De Marchi *et al*., unpublished) was analyzed by DIA MS and RNA-seq to validate our findings. This set was derived from a larger cohort of (109 samples), and tumors were selected based on availability of appearance data. Subtype classification and clinical and histopathological characteristics of breast cancer patient-derived specimens are reported in Figure 1C,D and Tables S1 and S2.

All tissues were collected from Lund University Hospital and affiliated clinics located in the Skåne region.

This study used primary breast tumor tissues under approval from the Ethical Review Board (Etikprövningsnämnden) with number DNR 2010/127.

### RNA and Protein Extraction

All breast cancer specimens and normal tissues were processed through an AllPrep (Qiagen) protocol for the lysis and extraction of RNA and proteins (Figure 1E). Except for the extraction of total protein content, all protocols were performed according to the manufacturer’s instructions (AllPrep RNA extraction kit). An amount of 20–30 mg of frozen tissues was cut and collected into tubes for downstream RNA and protein extraction. An adjacent piece, or imprint in cases where not enough tissue for embedding was available, was taken for microscopy and evaluation of the cancer content at the center performing RNA extraction. Steel beads (ID 79656, Qiagen) were added to each sample tube together with 400 μL of 1% β-mercaptoethanol in RLT buffer (Qiagen) and 2 μL of an antifoam agent (ID 19088, Qiagen). Tissue disruption was then performed in a TissueLyser LT (Qiagen) for 4 min at 50 Hz, after which a second volume of 400 μL of 1% β-mercaptoethanol in RLT buffer (AllPrep DNA/RNA Minikit, Qiagen) was added after the steel bead removal. Samples were then centrifuged at 14,000×*g* for 5 min. The supernatant was transferred to a new tube kept at −80 °C until RNA and protein extraction.

RNA extraction was performed as per manufacturer instructions using the AllPrep RNA Minikit (Qiagen). Flow-through of each column constituted the protein fraction, which was collected and stored at −80 °C prior to MS sample preparation.

### RNA Quality Control and Sequencing Analysis

The amount, concentration, and quality of the extracted RNA were tested using a Bioanalyzer 2100 instrument (Agilent Technologies, CA, USA), NanoDrop ND-1000 spectrophotometer (Thermo Fisher Scientific, MA, USA), or Caliper HT RNA LabChip (Perkin Elmer, MA, USA). All samples had a RNA integrity value (RIN) of 6.0 or higher.

RNA-sequencing analysis was conducted as previously described.^20^ Briefly, RNA concentration was measured in all AllPrep RNA eluates using a Qubit fluorometer following preparation with Qubit RNA HS assay (Thermo Fisher). A total of 100 ng of RNA input was then used for cDNA library preparation using a TruSeq Stranded mRNA NeoPrep kit (Illumina), according to the manufacturer’s instructions. Library cDNA concentration was then measured using a QuantIT dsDNA HS assay kit (Thermo Fisher), according to the manufacturer’s instructions. cDNA libraries were then denatured and diluted according to the NextSeq 500 system guide (protocol #15048776, Illumina). RNA-sequencing analysis was then performed on a NextSeq 500 (Illumina) sequencer generating paired-end reads of length 77 bp.

### Protein Quantitation and Digestion

Collected protein flow-throughs (FT) were subjected to protein precipitation for downstream protein content determination using a bicinchoninic acid assay (BCA, Thermo Fisher) and trypsin protein digestion. Protein extraction was performed by collecting the flow-through of RNeasy spin columns and by performing protein precipitation as follows: for every sample tube, three volumes of acetone were added and samples were incubated at −20 °C for 1 h. Samples were centrifuged at 14,000×*g* for 20 min, and the supernatant was removed. Protein pellets were washed twice with 200 μL of 95% ethanol solution for 10 min at room temperature. For FT samples, protein quantitation was performed by BCA assay after resuspension of the protein pellet in 1× PBS.

For whole tissue lysate (WTL) preparation, frozen tissues were prepared according to previously published protocols,^21^ with minor modifications. Briefly, 10 slices of 10 μm in thickness were cut for each frozen specimen and resuspended in ∼100 μL of ice-cold radioimmunoprecipitation assay (RIPA) buffer (150 mM sodium chloride, 1.0% NP-40 substitute, 0.5% w/v sodium deoxycholate, 0.1% w/v sodium dodecyl sulfate, 50 mM tris-(hydroxymethyl)aminomethane, pH 8.0) supplemented with Halt protease inhibitor cocktail (Thermo Fisher) and sonicated in a cooled bioruptor-type sonicator (Diagenode) for 15 min. Lysates were then centrifuged at 14,000×*g* for 20 min at 4 °C, and supernatants were transferred in a new tube. Protein concentration was measured by BCA assay (Thermo Fisher).

For downstream trypsin digestion, proteins were precipitated in acetone and washed in ethanol solution (as previously described^22^). Briefly, precipitated protein pellets were then resuspended 100 mM Tris (pH 8.0) buffer containing 100 mM dithiothreitol and 4% w/v sodium dodecyl sulfate and incubated at 95 °C for 30 min under mild agitation. Samples were then cooled to room temperature and diluted in 8 M urea in 100 mM Tris (pH 8.0) buffer for downstream protein digestion. Samples were then loaded on 30 KDa molecular filters (Millipore) and centrifuged at 14,000×*g* for 20 min. Filters with immobilized proteins were then washed with 100 μL of 8 M urea buffer and centrifuged at 14,000×*g* for 10 min. Filters with immobilized proteins were then incubated with 8 M urea buffer containing 50 mM iodoacetamide for 30 min in the dark. Filters were washed twice with 8 M urea buffer followed by two washes with 50 mM triethylammonium bicarbonate buffer (pH 8.0). Proteins were then digested with trypsin (enzyme:protein ratio of 1:50) at 37 °C for 16 h under agitation (600 RPM). Filters were then centrifuged at 14,000×*g* for 20 min to retrieve tryptic peptides.

For DDA MS analysis (sample set 1), a total of 50 μg of the protein content was digested for each sample followed by strong anion-exchange fractionation following previously described protocols.^13^ Briefly, digested peptides were dried and resuspended in Britton and Robinson universal buffer (20 mM phosphoric acid, 20 mM boric acid, and 20 mM acetic acid in ultrapure water; BRUB, pH 11) and loaded on strong anion-exchange (SAX; six stacked layers; 66888-U, Sigma) stage tips. SAX filter-containing tips were put on top of C18 (three stacked layers; 66883-U, Sigma) stage tips, and peptides were eluted with 100 μL of pH 11 BRUB buffer. SAX stage tips were then transferred onto new C18 tips, and peptides were eluted serially at different pHs: 8, 6, 5, 4, and 3. C18 tips were then collected, washed with 0.1% formic acid (FA) in ultrapure water, and eluted with 100 μL of a solution containing 0.1% FA and 80% acetonitrile in ultrapure water. To eliminate any possible remaining contaminants, eluates were dried and subjected to SP3 peptide purification (as described in Hughes *et al*.^23^). Briefly, 2 μL of SP3 beads (1:1 ratio of Sera Mag A and Sera Mag B resuspended in ultrapure water, Sigma) was added to dried peptides and incubated for 2 min under gentle agitation. A volume of 200 μL of acetonitrile was then added, and samples were incubated for 10 min under agitation. Sample vials were then placed on a magnetic rack and washed again with acetonitrile for 10 min. Elution was performed by adding 200 μL of 2% dimethyl sulfoxide in water to the beads–peptides mixture and incubating them for 5 min under agitation. Supernatants were then collected, dried, and stored at −80 °C until MS analysis.

For downstream DIA MS analysis (sample sets 1 and 2), a total of 10 μg of protein was digested as previously mentioned, omitting SAX stage tip-based fractionation. Solid-phase extraction was performed using the SP3 method, as aforementioned.

### Mass Spectrometry Analysis

Global proteome DDA MS analysis was performed on a Q-Exactive Plus (Thermo Fisher) mass spectrometer (sample set 1). Around 1 μg of tryptic peptides from fractionated samples was separated on an RP-HPLC EasySpray column (ID 75 μm × 25 cm C18 2 μm 100 Å resin, Thermo Fisher) coupled to an EASY-nLC 1000 liquid chromatography system (Thermo Fisher).

For DDA analysis of SAX-fractionated samples, peptides from each fraction (*n* of fractions: 6) were eluted in a 90 min gradient (flow: 300 nL/min, mobile phase A: 0.1% formic acid in H_2_O, mobile phase B: 99.9% acetonitrile and 0.1% formic acid). The chromatographic gradient was run as follows: 5% B for 5 min, 5–30% B in 85 min, 95% B for 10 min. The 15 most abundant peaks from the MS scan (resolution: 70,000 at 200 *m*/*z*) were selected and fragmented by higher energy induced collision dissociation (HCD, collision energy: 30). Dynamic exclusion was enabled (window: 20 s). The AGC target for both full MS and MS/MS scans was set to 1 × 10^6^. Precursor ions with intensity above 1.7 × 10^4^ were selected for MS/MS scan triggering.

For DIA MS analysis (sample sets 1 and 2), a Q-Exactive HF-X (Thermo Fisher) mass spectrometer was employed. Unfractionated samples were eluted in a 120 min gradient (flow: 300 nL/min, mobile phase A: 0.1% formic acid in H_2_O, mobile phase B: 80.0% acetonitrile and 0.1% formic acid) on a Q-Exactive HFX (Thermo Fisher) instrument coupled online to an EASY-nLC 1200 system (Thermo Fisher). Digested peptides were separated by RP-HPLC (ID 75 μm × 50 cm C18 2 μm 100 Å resin, Thermo Fisher). The gradient was run as follows: 10–30% B in 90 min; 30–45% B in 20 min; 45–90% B in 30 s, and 90% B for 9 min. One high-resolution MS scan (resolution: 60,000 at 200 *m*/*z*) was performed and followed by a set of 32 DIA MS cycles with variable isolation windows (resolution: 30,000 at 200 *m*/*z*, isolation windows: 13, 14, 15, 16, 17, 18, 20, 22, 23, 25, 29, 37, 45, 51, 66, 132 *m*/*z*; overlap between windows: 0.5 *m*/*z*). Ions within each window were fragmented by HCD (collision energy: 30). The automatic gain control (AGC) target for MS scans was set to 1 × 10^6^ for MS and MS/MS scans, with ion accumulation time set to 100 and 120 ms for MS and MS/MS, respectively (Table S3).

### DDA MS Data Processing

DDA-derived RAW files were analyzed using MaxQuant (v1.6.0.16). MS spectra were searched using the Andromeda built-in search engine against the Uniprot-Swissprot human proteome database (version download 2017.06.12). Label-free quantification (LFQ) and match between run options were enabled. The chosen protease was trypsin. Identification of peptides resulting from missed cleavages was allowed. Fixed modifications: carbamidomethylation of Cys residues. Precursor ion tolerance: 20 and 4.5 ppm for first and main searches, respectively. Variable modifications: acetylation of the N-terminal residue, oxidation of Met residues. Proteins were then filtered for false discovery rate (*q*-value <1%), reverse sequences (excluded), contaminants (excluded), and identification of unique peptides (at least one unique peptide per protein). LFQ intensities were then Log2 transformed and protein-level scaled prior to statistical analysis.

### DIA MS Data Processing and Spectral Library Generation (MakeGTL Workflow)

The workflow used in the spectral library generation for the DIA search is described in Figures S1A and S2A. This computational pipeline uses the DDA raw data by first employing MaRaCluster (v0.05.0, build date: Apr 16, 2018 21:04:32) to cluster the spectra and then generating consensus spectra from the clustering using a 5% clustering *p*-value cutoff. Other parameters in MaRaCluster were set to default. In the next step, the consensus spectra of the selected clusters were searched against the protein sequence database (Uniprot-Swissprot version download 2017.06.12 for general protein quantification) using Comet (v2017.01 rev. 4^24^), and the resulting peptide spectral matches (PSMs) were scored by Percolator (v3.02.1, build date: Aug 13, 2018 15:50:58^25^). The resulting scored PSMs were then processed using an in-house built python script that selected (for each peptide) the spectral match with the best *q*-value smaller than 10%. Subsequently, our script extracted transitions from each spectrum by matching peaks to theoretical ion masses within 1 ppm (only y and b ions were considered here since these were the most commonly observed ions). The resulting output consisted of an OpenSWATH compatible generic transition list (GTL) in tsv format. We applied this workflow to three sets of raw DDA files (parameter sets were similar to those of MaxQuant, where applicable): the datasets generated in this study are the Tyanova^13^ and Bouchal^14^ datasets.

### Iterative RT Peptide Selection and Quantification in DIA Analysis (DIAnRT Workflow)

To select a set of internal iRT peptides (i.e., peptides that are endogenously present in each sample run), OpenSWATH was run without iRT peptide input to extract the best peptide candidates (i.e., eluting within peptide-dense chromatogram regions) to be used as iRT peptides for the next iteration (Figure S1B). Naturally occurring peptides allow for high-accuracy alignment of feature, increasing identification sensitivity.

We used a Python script to extract from the resulting OSW files a set of peptides that were detected closest to their library retention time (at most 10 min). These were then additionally filtered based on peak width (i.e., less than 16.5 s at the base) and intensity (i.e., at least 1 × 10^5^). Peptides detected in less than 20 samples were discarded, and the remaining set of peptides was randomly subsampled to not more than 100 peptides per retention time (RT) bin when splitting the DIA gradient into 20 equally sized RT bins. This set of peptides was then used in the next step to fit a lasso iRT model with OpenSWATH (with parameters related to the DDA searches, where applicable), and the results were again processed to extract the best-fitting, sharp-peaked peptides that were found in many samples. Each set of output files was scored using PyProphet (v2.0.1) by merging a subsample of 5% of the peptide-spectral matches (PSM) from each sample and scoring the merged dataset (scoring level: MS1–MS2). The resulting model (build on a representative sample comprised of 5% from each individual sample) was then back-propagated to the individual samples and used for their scoring. The scored samples were then passed to the feature alignment.py script for TRIC alignment.^26^ After five iterations, the number of proteins identified with this method seemed to stabilize (4219, 4281, 4298, 4302, and 4301, respectively, using the library generated from our DDA data), and for this, we chose the last iteration results as our dataset for further analysis.

After requantification, 28,746 peptides covering 4936 proteins were quantified. These were scaled by per-sample median intensity to account for sample-level differences. A Log2-transformed, mean-centered, and standard deviation-scaled version of this matrix was generated both on the peptide and protein levels. For protein summarization, we first selected for each protein the larges subgroup of at least three peptides that had a Spearman correlation of at least 0.7 among them, summed those peptides intensities, and applied the Log2 transformation, scaling, and centering on this summed intensity. We applied the same workflow to all three generated libraries (our own, Tyanova, and Bouchal) individually.

### Combining Results from Multiple DIA Library Searches

For general peptide quantification, we employed three libraries (Subtypes, Tyanova, and Bouchal) in the DIAnRT workflow to create three respective sets of peptide quantifications, which were combined before PyProphet^27^ scoring. For each peptide in the superset of quantified peptides from all three libraries, *p*-values were selected according to the following rules:

1. If the peptide is quantified in our own library, we used that *p*-value.
2. If the peptide is quantified in only one library, we used that *p*-value.
3. If the peptide is quantified in both the Tyanova and Bouchal libraries, but not in our library, we used the quantification with the lower *p*-value.

Peptide quantifications were then combined into a single table and processed with PyProphet for *q*-value calculations followed by feature alignment between DIA runs and requantification based on the alignment.

### RNA Data Processing

The demultiplexed RNA-Seq reads were aligned to the GRCh38 human reference genome using a STAR aligner (v020201) with an overhang value of 75 to match the read length. Subsequently, we employed the standard GATK analysis pipeline including duplicate removal, indel realignment, and base quality score recalibration (GATK v3.7-0-gcfedb67).

The resulting bam files were processed using the DESeq2 R/Bioconductor package (version 1.22.2) by first generating per-gene read counts mapping to the GRCh38 GTF file from Ensembl version 95 using the *summarizeOverlaps* function in “Union” mode so to count reads that uniquely mapping to exactly one exon of a gene. After discarding genes with no counts in any of the samples, DESeq analysis was performed with the ER status (i.e., ER positive and ER negative) as the explanatory variable in the model followed by log-fold-change shrinkage. A separate DESeq analysis was also performed using the mammographic appearance (i.e., spiculated and nonspiculated tumors) status as the explanatory variable.

For DTU detection (the computational workflow is shown in Figure S1C), we employed RNA-guided spectral libraries. The BANDITs^28^ workflow was employed to analyze the RNA-seq data and to determine a set of genes with differential transcript usage in the comparison of ER-positive and ER-negative samples.

To verify DTUs at the proteome level, an isoform-aware spectral library was generated from the Ensembl GRCh38 human proteome by *in silico* tryptic digestion of all protein isoforms found in this database and the determination for each peptide the set of protein isoforms it matched to. For each unique combination of isoforms, all matching peptides of at least length five amino acids were concatenated to create a mock protein sequence specific to that combination of isoforms. Using the resulting FASTA file and our DDA data, an isoform-aware spectral library was created, where each detectable peptide was matched to a set of protein isoforms identified by their Ensembl protein IDs. The DIAnRT workflow was employed on this library to quantify peptides in an isoform aware fashion from our DIA data (i.e., by reusing the set of RT peptides generated in the iterative DIA quantification process described above). Quantified peptide intensities of those proteins that matched to genes with significant differential transcript usage were overlaid onto those determined by the BANDITs workflow on the RNA-seq data.

For SNV/SAAV evaluation (Figure S1D), SNV calls were derived out of the aligned RNA-seq reads using the h5vc R/Bioconductor package with the callVariants function, requiring at least 2 reads supporting the variant and at least 10 reads total coverage. Similar to previously published workflows employed for the analysis of DDA datasets,^29^ we annotated the SNVs using the Ensembl variant effect predictor and filtered the SAAVs to retain only those events that modify the amino acid sequence of the affected protein.

By using the set of SAAV calls obtained from the RNA-seq data, we generated (for each SAAV) its derived protein sequence (DIA level only) and used *in silico* digestion to determine the resulting set of tryptic peptides. By discarding all peptides that also arose from the unmodified reference sequence, a set of peptides that specifically identify each SAAV was determined (typically only one peptide, except where SAAVs generated new tryptic peptides). From these results, a FASTA file containing the concatenated peptide sequences that identify each SAAV was created and subsequently used as an input within the MakeGTL workflow to create a SAAV library for downstream SAAV quantification.

### Immunohistochemistry

Formalin-fixed and paraffin-embedded (FFPE) tissues were cut into 3–4 μm sections and put on FLEX IHC microscope slides (K8020, DAKO). Slides were heated at 60 °C for 60 min and deparaffinized in xylene (2 × 10 min). Rehydration was performed in decreasing concentrations of ethanol (100% ethanol: 1 × 5 min, 95% ethanol: 1 × 5 min) followed by rinsing in distilled water. The immunohistochemical (IHC) staining for KI67 was performed using an Autostainer Plus (DAKO) instrument. Antigen retrieval was performed on a PT-LINK (Agilent) instrument using the EnVision FLEX target retrieval solution (pH 9, dilution: 1:10) at 98 °C for 20 min. Slides were stained by incubating the primary antibody (Ki67:clone MIB-1, M7240, Agilent Technologies) at the following dilution: 1:200 (temperature: RT, time: 30 min). The antibody–antigen complex was visualized using the EnVision FLEX DAB detection kit (K801021-2, Agilent Technologies) and counterstained with Mayer’s hematoxylin (S3309, Agilent Technologies). Stained slides were dehydrated in increasing concentrations of ethanol (95% ethanol: 1 × 3 min, 100% ethanol: 1 × 3 min), followed by xylene (2 × 5 min). Cover glasses were mounted using a Coverslipper DAKO (Agilent Technologies), and slides were left to dry prior to staining evaluation.

### Immunohistochemical Staining Analysis

All HE evaluations and IHC staining scorings were evaluated and performed by a trained pathologist (GP). For KI67, only the percentage of positive tumor cells was assessed. The KI67 status was defined by the current standard of practice in Southern Sweden (positivity cutoff: ≥ 30).

### Statistical and Pathway Analyses

In the analyses of the tumors included in sample set 1, proteins with less than 30% missing observations (<30% missing data) in the DDA set were included. This resulted in a list of 2796 proteins. Welch-corrected *t* test was performed to assess significant differences followed by Benjamini–Hochberg *p*-value adjustment as multiple test correction.

In our correlation analyses between transcript and protein abundances, we employed Spearman correlation to calculate both the correlation coefficient and *p*-value. To assess whether specific protein clusters were affected by different mRNA-protein correlation distributions, all proteins were annotated with GOBP terms; the distribution of correlation coefficients of each GOBP annotation was then tested against the background (i.e., all proteins) by *t* test followed by Benjamini–Hochberg *p*-value adjustment. The selected adjusted *p*-value cutoff for GOBP annotation was 0.15.

In all the analyses for differential pathway enrichment between ER statuses (ER positive *vs* ER negative), we performed gene set enrichment analysis (GSEA,^30^ database: Hallmarks v5.2, permutation type: gene set, scoring: weighted, metric: *t* test, other parameters were kept at default settings, significance cutoff: FDR < 0.25) on RNA, DDA (FT subset only), and DIA data layers. Input data tables were filtered as follows: RNA (no filtering), DDA (<30% missing observations), and DIA (<30% missing observations). Enrichment scores of the top50 (or all if <50)-significant (i.e., by *q*-value) pathways were then plotted for each data layer.

To define protein co-regulation clusters in our DDA and DIA datasets, we generated Spearman correlation-based matrices for the ER-positive and ER-negative groups. Using the elbow method, the minimum number of clusters was then defined for each ER-status sample group. Significant pathway annotations (FDR < 0.05) from the Panther over-representation test (database: GOBP complete, http://www.pantherdb.org/) were used to annotate each cluster. Distances (metric: Euclidean) between the clusters based on GOBP annotations were then calculated to subsequently merge highly similar clusters employing a second iteration of the elbow method.

Plots have been generated in R v3.6.1. Quantitative proteomic information of all datasets is available as Tables S4–S6.

## Results

### Generation of a Proteogenomic Data Set for Breast Cancer

In this study, we selected 21 tumor samples (sample set 1) from a larger study^18^ with associated mammography images, hormonal receptor status, and clinical histopathological information. A second set of 24 tumor tissues was selected based on availability of RNA-seq, MS data, and mammographic imaging information (sample set 2, Figure 1A,B). In these sets, ER-status frequency resembled the one of the general population (i.e., ∼70% ER positive, ∼30% ER negative): 13 (61.9%, set 1) and 17 (70.8%, set 2) tumors were positive to ER, while only 8 (38.1%) and 7 (29.2%) were ER negative in sample sets 1 and 2, respectively. In total, seven patients from sample set 1 and four patients from sample set 2 had tumors with a spiculated appearance (Figure 1C and Tables S1 and S2). While spiculated tumors were largely ER positive, no significant association was found between these characteristic (Figure 1D).

RNA was extracted from all samples followed by RNA sequencing (RNA-seq). In addition, proteins were extracted using ALLPrep (flow-throughs, FT) and standard tissue homogenization (whole tissue lysate; WTL, sample set 1 only) followed by trypsin digestion. The tryptic peptides were analyzed directly using DIA MS and fractionated using strong anion exchange (SAX) followed by DDA MS analysis (Figure 1E).

In the next step, we used the RNA, DDA, and DIA data to develop an improved DIA MS-based proteogenomic workflow for breast cancer (Figure S1A,B). First, we developed a DIA library creation method that improves signal-to-noise characteristics of the MS2 spectra through spectral clustering. We applied the method to three separate datasets as follows: our own DDA data (FT and WTL combined for increased peptide identifications and protein coverage, Figure S3; De Marchi), as well as the DDA data from Tyanova *et al.* and Bouchal *et al.*(13,14) The three assay libraries covered 71,152 (this study: De Marchi), 61,282 (Tyanova), and 41,018 (Bouchal) peptide groups (peptide + charge), which mapped to 9953, 9678, and 6971 proteins, respectively. Second, we integrated transcript sequence information and single-nucleotide variants from RNA-seq into the workflow used for library generation (Figure S1C,D). In this way, a transcript-aware DIA library was created covering 89,538 differential transcript usage (DTU) sites matching to 43,910 different transcripts and 12,488 genes. In addition, we predicted SAAVs by SNV calling on the RNA-seq data to create a SAAV aware DIA library composed of 1025 RNA-guided transitions for 74 SAAVs. Third, we implemented an iterative process for selecting intrinsic retention time (iRT) peptides to improve retention time alignment (Figure S1A,B).^31^

For quantitative analysis, we used the three spectral libraries and improved retention time alignment to increase the peptide identification rates from the DIA data. The results from the three libraries were combined to create a superset, in which peptide *p*-values were conditionally selected for downstream *q*-value determination, feature alignment, and requantification (Figures S1A,B and S2A), which resulted in the quantification of 28,746 peptides matching 4936 proteins (Figure S2B). Upon comparing the proteomic layers, we observed that the DDA layer comprised a higher number of peptide and protein identifications (DDA total peptides/proteins: 60,857/7106, DIA: total peptides/proteins: 28,746/4936), though DIA MS data displayed a higher percentage of consistent identifications across samples (DDA-consistent peptides/proteins: 1914/1473, DIA-consistent peptides/proteins: 18,218/3905, Figure S4). This is likely due to the inherent difference in data acquisition between the two methods, where DDA employs a stochastic approach to select precursor ions to perform fragmentation spectra acquisition, while DIA fragments every precursor ion in the retention-time plane. This has resulted in a sparser quantitative dataset at the DDA level.

The 28,746 identified peptides were used for protein quantification and to detect SAAV and DTU, while the RNA-sequencing information provides data related to SNV, DTU, and RNA abundance. The data layers were then integrated and compared to extract information of relevance for spiculation and receptor status and frequency of DTU, to determine the degree of corroboration or discrepancy between protein- and RNA-quantitative information and to identify SAAV-specific peptides.

### Comparison between RNA and Proteomic Data Layers

**Figure 2:**
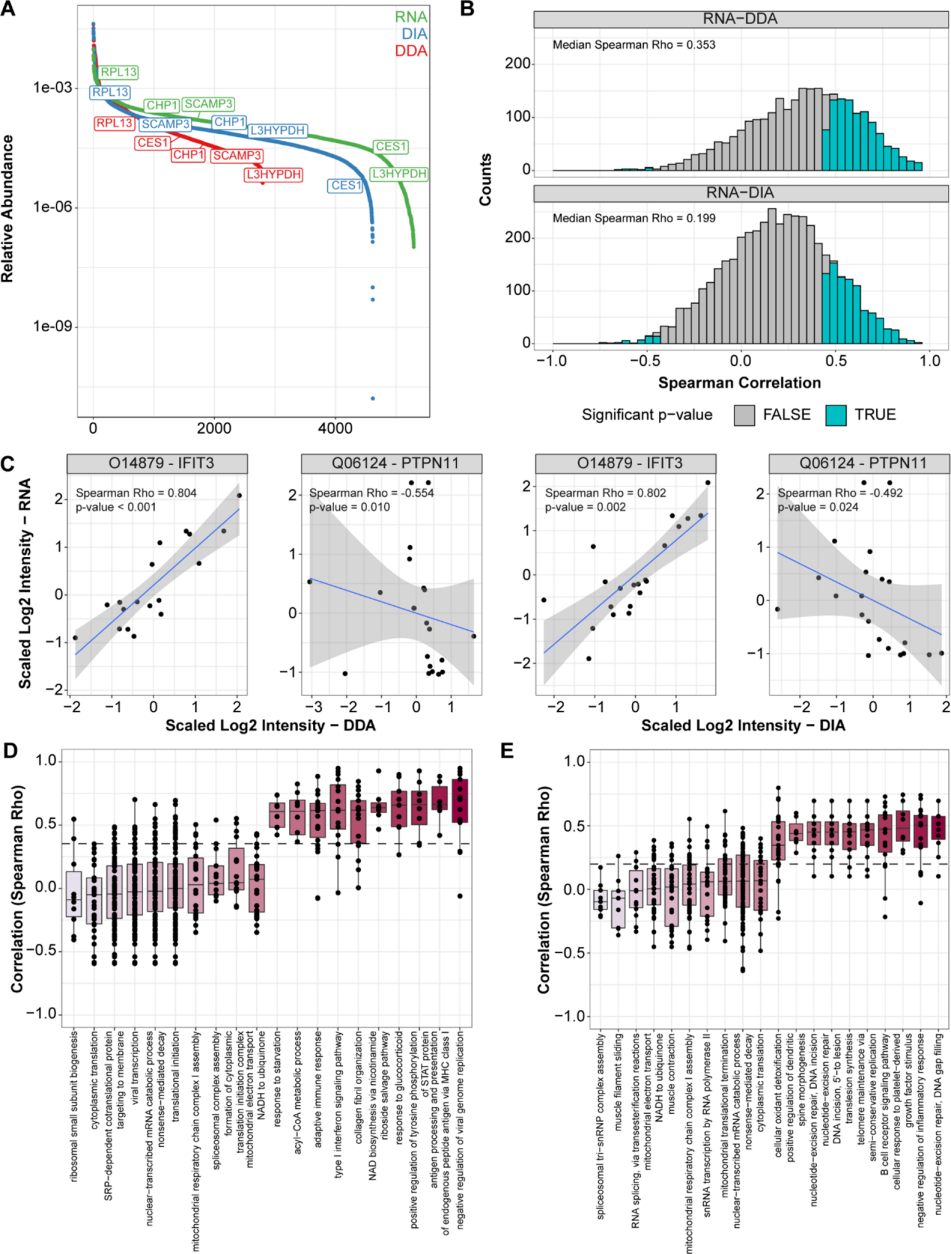
Overall comparison between transcriptomic and proteomic data layers. Panel (A) displays the dynamic range (presented as relative abundance over total signal) of transcript and protein intensities of matching identifications in our RNA (green), DDA (red), and DIA (blue) MS data (examples of transcript–protein pairs displaying similar abundances across data layers are labeled). Distributions of Spearman correlations between matching transcript and protein (DDA: top, DIA: bottom) abundances are displayed in panel (B) (gray: nonsignificant, light blue: significant), while examples of consistent positive and negative correlation between protein levels (DDA and DIA) and RNA abundance are depicted in panel (C). Panels (D) and (E) display the distribution of transcript–protein correlations for significant (*q*-value < 0.15, see Experimental Procedures for details) GOBP pathways out of our DDA and DIA MS analyses, respectively. Color gradient is representative of the low (pink) and high (dark red) median transcript–protein correlation for each GOBP term. Acronyms: DDA: data-dependent acquisition, DIA: data-independent acquisition, ER: estrogen receptor, GOBP: gene ontology biological process.

Based on the combined proteogenomic data set, we compared the overall quantitative measurements between the transcriptomic and proteomic data sets in sample set 1. Transcripts were matched to their respective protein products to assess the dynamic range. The RNA data displayed a flatter dynamic range slope, which might relate to technological differences between gene and protein quantitation technologies, where RNA-seq achieves a more even gene quantitation across the transcriptome. Alternatively, this difference might be related to the fact that mRNA data does not accurately reflect post-translational processes, such as ubiquitination and degradation processes, which operate exclusively at the protein level (Figure 2A). The proteomic data sets displayed a wider dynamic range, although the transcript and protein abundances were often located in similar quantiles across each sigmoid. To systematically compare transcriptomic and proteomics data sets, we calculated transcript–protein correlations for all detected transcript–protein pairs (Table S7). We observed a relatively wide range of correlations (Spearman Rho range: −0.752 to 0.956, Figure 2B), corroborating findings from a previous work.^12^ Overall, more than 75% of the transcript–protein pairs showed positive correlation coefficients and agreement/disagreement between the RNA data and the protein was often consistent for both the DDA and DIA layers (Figure 2C). In contrast, a minority of transcript–proteins pairs displayed negative correlations, such as RBM39 and EXOC3. Only a handful of these negative correlations were significant after *p*-value adjustment, suggesting that anticorrelating transcript–protein pairs might result from technical variation (Figure S5). We could confirm this observation in sample set 2 with a similar distribution of correlation coefficients (Spearman Rho range: −0.667 to 0.936, Figure S6A). In this samples set, we also observed relatively few significant negative correlations (Figure S6B,C. Of note, the DDA and DIA data layers displayed significant agreement in their relation to RNA (Figure S7).

The variability of transcript–protein correlations might be related to specific protein subclasses and biological pathways, as recently shown in another BC study.^12^ Analysis of enriched gene ontology pathways confirmed these observations, showing that the degree of correlation was strongly related to pathways such as RNA splicing or inflammatory response (Figure 2D,E). Altogether, these results suggest that factors that alter protein abundances, such as post-translational modification and protein degradation, have a larger impact on certain protein classes. This is likely related to cellular regulation of internal processes and a response to external stimuli, such as mitophagy.^32^

### Pathways Related to the Estrogen Receptor Status and Mammographic Appearances

**Figure 3:**
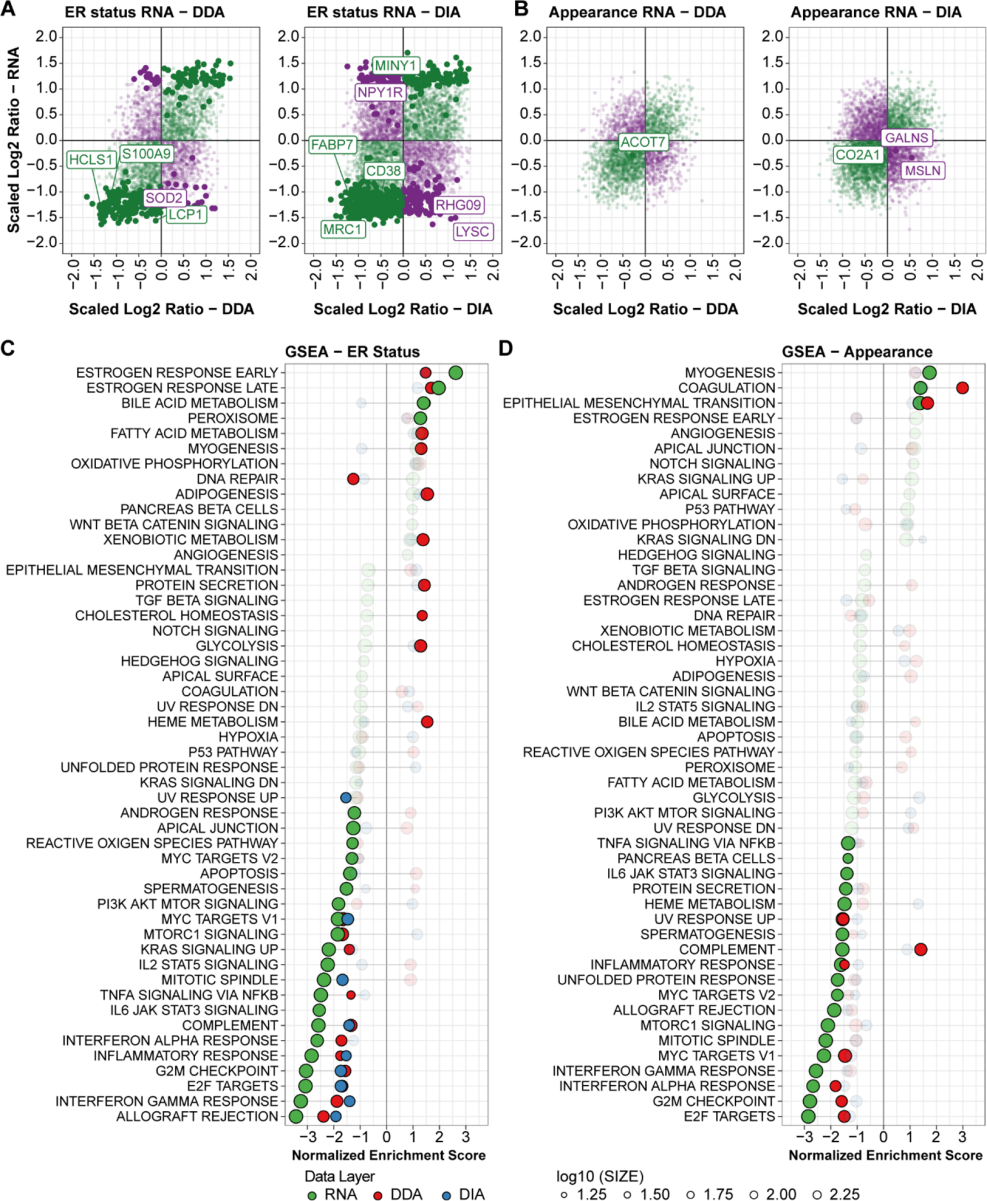
Comparison between transcriptomic and proteomic data in the context of the estrogen receptor and appearance statuses. Panels (A) and (B) display all transcript–protein pairs scaled Log2Ratios for the ER status (A) and appearance ((B); DDA: left, DIA: right). Significant differential expression at the RNA level is marked by full dots and in bigger size; concordance and discordance between RNA and protein layers are shown in green and purple, respectively). Most significant genes (top 5% quantile) are shown in labels. GSEA analyses were performed on all data layers (RNA, DDA, and DIA) for ER and spiculation statuses using the Hallmark database. Pathways are ranked based on the RNA-level enrichment score. Panel (C) displays the overlap of GSEA analyses for the ER status, while panel (D) shows the results of analysis of appearance features (i.e., spiculation *vs* no spiculation). Significant pathways in each data layer (RNA: green, DDA: red, DIA: blue) are marked in full color, while transparent ones did not pass the false discovery rate (FDR < 0.25) cutoff. Positive scores mark enrichment in ER-positive and spiculated tumors, respectively, while negative scores define enrichments in ER-negative and nonspiculated samples. Acronyms: DDA: data-dependent acquisition, DIA: data-independent acquisition, ER: estrogen receptor, FDR: false discovery rate, GSEA: gene set enrichment analysis.

The combined proteogenomic data set provides new possibilities to investigate differences between clinically relevant tumor groups such as receptor status and mammographic appearances such as spiculation. For this reason, we stratified the breast cancer discovery sample set according to ER status and mammographic appearance (Figure 1B) and filtered for differentially expressed genes (RNA level) for each group comparison (Tables S8 and S9). Regulation was concordant for most significantly regulated transcript–protein pairs in both the ER status and mammographic appearance comparison (Figure 3A,B, full green dots), with only a small subset (∼18–30%) of significant transcript–protein pairs displaying an opposite regulation pattern (Figure 3A,B, full purple dots). The magnitude of regulation was substantially higher in the ER status group when compared to tumor appearance. ER has a large impact on cell proliferation pathways and is connected to different cell lineages, i.e., basal and luminal, which is a likely explanation to the substantial differences observed between ER-positive and ER-negative tumors.

We annotated the differentially significant genes/proteins from ER and mammographic appearance status comparison using the Molecular Signatures Database to assess whether the RNA/protein abundance discrepancies belonged to specific functional groups. Several of the differentially expressed transcripts were enriched in immune pathways (e.g., allograft rejection, Figure S8A,B), and the RNA–transcript correlations had significant positive correlations independent of pathway (Figure S8C,D). These results suggest that the transcript–protein abundance discrepancies within the subset of significant transcript–protein pairs are not related to particular functional groups.

Next, we investigated whether the combined transcriptomic and proteomic data could provide complementary information in terms of pathway enrichment within tumor subgroups.^11^ We performed gene set enrichment analysis (GSEA) on the three data sets for the ER status and mammographic appearance (Figure 3C). As expected, the estrogen early and late response gene networks were the pathways with the highest positive enrichment scores for the ER status. For spiculation, the pathways with the highest enrichment score were myogenesis, coagulation, and epithelial–mesenchymal transition (EMT). To rule out tissue morphological features, we evaluated variability in tumor cellularity, number of fibroblasts and red blood cells, and Ki67 staining between ER and spiculation status tissues (Figure S9A,B), though no significant differences were observed. In contrast, immune response (e.g., allograft rejection, interferon response) and cell cycle (e.g., G2M Checkpoint) pathways were found enriched in nonspiculated tumors. Pathways enriched at the RNA and protein level often displayed similar levels of enrichment. In addition to this, the proteomic and transcriptomic data layers provided complementary information (i.e., common enrichment) regarding translation (e.g., E2F targets) and immune response (e.g., interferon gamma response) pathways, where significant changes in metabolic networks (e.g., fatty acid metabolism) were only detected at the protein level. Collectively, these findings hint at the fact that alterations in specific pathways or class of proteins might only be detected at the protein level.

To confirm these findings in an independent sample cohort, we performed differential expression and GSEA analyses on the RNA-seq and DIA MS data in sample set 2. Here, we observed that RNA and protein maintained a high level of agreement for differentially expressed genes (Figure S10A,B). On top of this, we could confirm the enrichment of ER-related pathways in the ER-positive tumors and immune signatures in the ER-negative tumors (Figure S10C). In addition, the analysis of this set confirmed the significant enrichment of transcription-related pathways, such as the E2F targets and EMT gene sets in the nonspiculated and spiculated tumor groups, respectively (Figure S10D).

**Figure 4:**
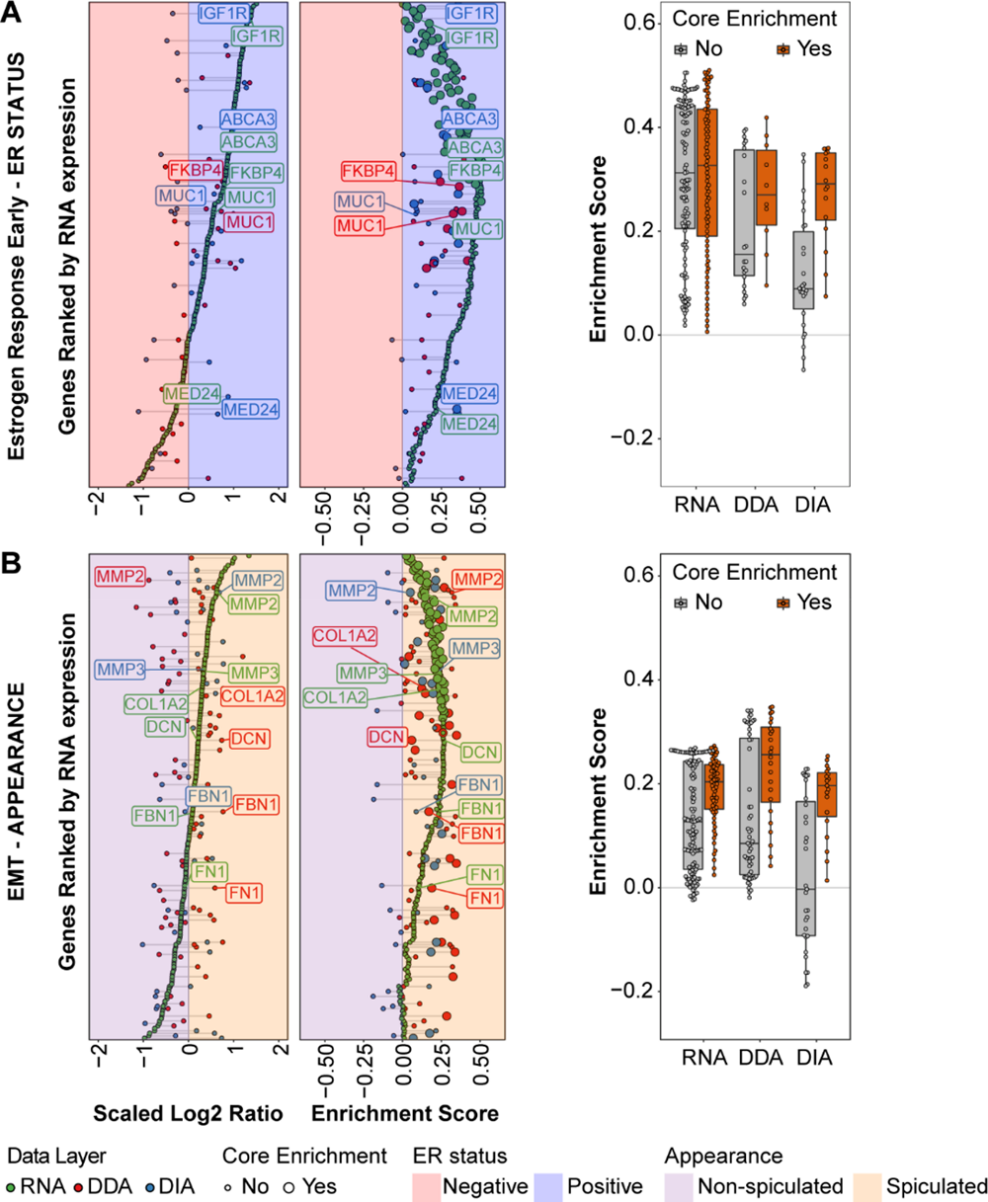
Pathway-level comparison of transcript–protein pairs. The figure displays transcript–protein-wise comparison within significant pathways out of GSEA analyses for the ER status (estrogen response early, (A)) and appearance (epithelial mesenchymal transition, (B)). Left panels display Log2Ratios of each transcript/protein (ranked by RNA expression) between ER-positive/negative and spiculated/nonspiculated tumors, while center panels display the corresponding enrichment scores in each data layer (RNA: green, DDA: red, DIA: blue). Right panels show distribution of enrichment scores for core-enriched (red) and noncore-enriched (gray) transcript/proteins. Left and center plots background color denotes enrichment in ER-positive (blue) and ER-negative (red) groups and spiculated (orange) and nonspiculated (purple) tumor groups. Abbreviations: DDA: data-dependent acquisition, DIA: data-independent acquisition, ER: estrogen receptor, FDR: false discovery rate, GSEA: gene set enrichment analysis.

Of the pathways with the highest enrichment score (sample set 1), we selected two for further analysis. The most enriched pathway in ER-positive tumors was the estrogen response early gene set (Figure 4A), which includes genes involved in signal transduction processes and cell differentiation (e.g., IGF1R and MUC1), as well as transcription factor-associated proteins such as MED24. Here, enriched transcripts and proteins included both ER-bound proteins (e.g., FKBP4) and genes activated downstream of ER transcriptional activity (e.g., ABCA3), which relate to downstream activation of breast tissue hormone-dependent proliferation mechanisms.

Conversely, the most enriched pathway in spiculated tumors was EMT, which is constituted by a high number of extracellular proteins, suggesting a marked interaction between the cancer mass and its surrounding tissue within spiculated tumors. Proteins dedicated to extracellular matrix remodeling (e.g., MMP2) and organization (e.g., FBLN5) as well as molecules involved in the induction of a mesenchymal cell state (e.g., FN1, Figure 4B) were enriched both at the RNA and protein levels. This suggests reprogramming of the tumor front for tissue invasion. Given the fact that spiculae protruding from the tumor mass are signs of cancer spread into the surrounding normal tissue, it is likely that remodeling of the extracellular matrix takes place in spiculated cancers.

Our results confirm previously characterized properties of ER-negative tumors such as increased immunogenicity and genomic instability,^33^ as well as pinpointing the discrepancy between RNA and protein abundances. In addition to this, we shed light on, so far, uncharacterized tumor aspects underlying spiculated cancer appearance, where the stroma is rearranged around the tumor mass to facilitate invasion. Conversely, the processes operating in nonspiculated cancers seems to revolve around cell proliferation pathways, thus indicating that mammographic appearance features might be related to different cell fates (e.g., proliferation *vs* invasion).

### Protein-Level Translation of the Splice and Amino Acid Variants

As outlined above, the proposed workflow facilitates proteogenomic analyses of DTU and SNV/SAAV expression. For the DTU analysis, the Bayesian analysis of differential splicing (BANDITs)^28^ workflow was employed to define differentially expressed transcripts belonging to the same gene in our RNA dataset. DTU features were then integrated at the library level by integration into our spectral library generation and search workflow (Figure S1 and Experimental Procedures). The analysis between ER-positive and ER-negative tumors generated 539 significant cases of differential transcript usages belonging to 451 genes (RNA-level FDR cutoff: 0.03). Pathway enrichment analysis revealed no significantly enriched pathway (ReactomePA R/Bioconductor package in version 1.30.0^34^).

The same analysis was performed for the mammographic appearance group comparison, which detected 63 differentially used transcripts in 55 genes (FDR 0.03). Here, the Reactome pathway enrichment analysis returned only one enriched network: “endosomal/vacuolar pathway” (*q*-value = 0.032), which relates to MHC-1 antigen presentation and adaptive immunity. However, given the relatively sparse input data (i.e., 55 genes, see above), these results need further investigation. On top of this, functional assays would be required to confirm the role of the adaptive immune system in its relation to tumor mammographic appearance.

**Figure 5:**
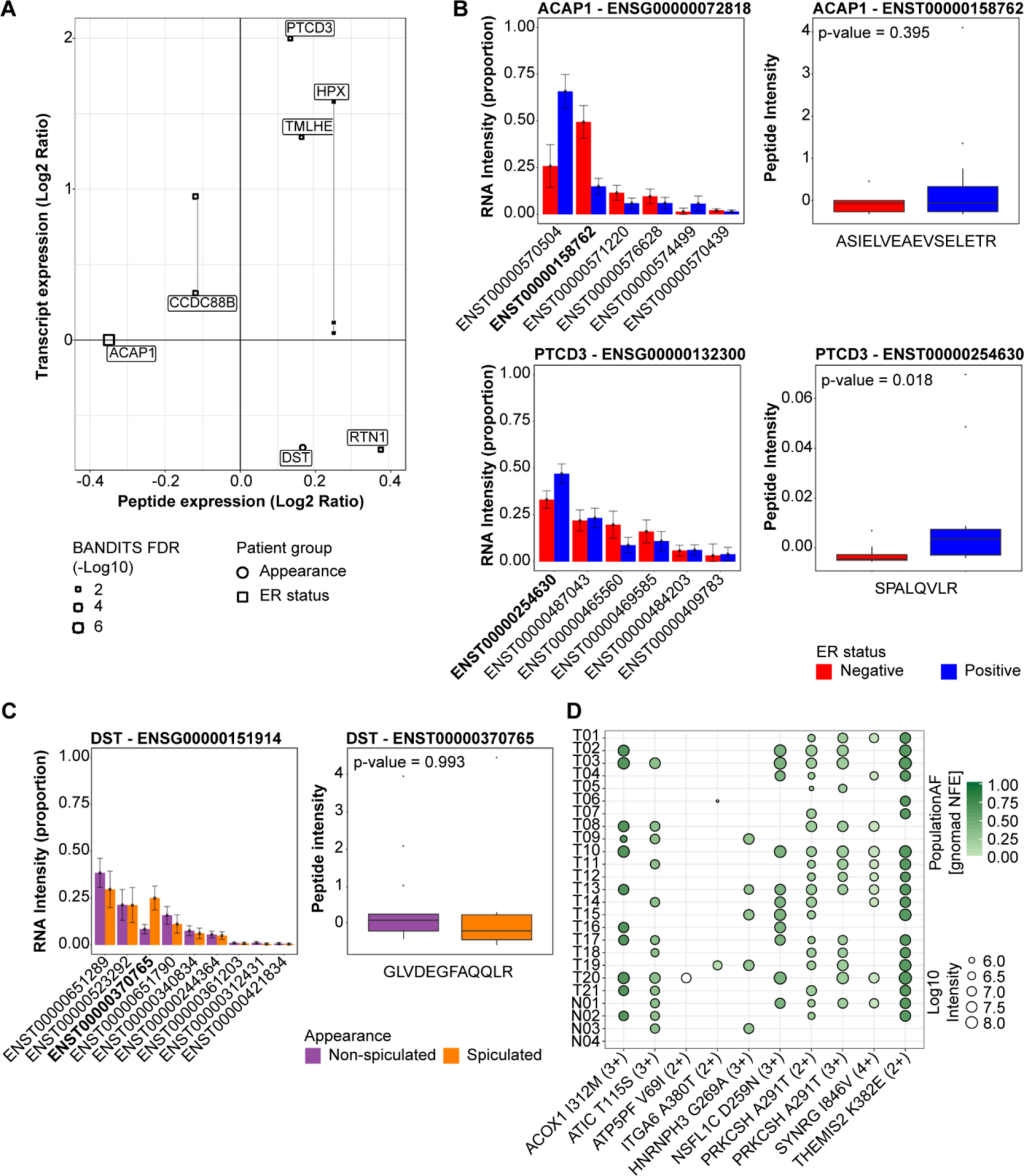
Evaluation of differential transcript usage and single amino acid variant detection at the proteomic level. We employed transcriptomic data information to search our DIA data for DTU (A–C) and SAAVs (D, E). For DTU analysis, we employed the BANDITs workflow to define transcript differential expression to then generate an isoform-aware spectral library with which to search our DIA MS data. Panel (A) displays detected DTU at the protein (DIA MS) level and their expression compared to transcript levels. Examples of transcript (left) and (when detected) their specific peptide (right) expression are shown in panel (B) (ER status) and (C) (appearance). *t* Test *p*-value is shown for box-plots (peptide level). For SAAV detection, nonsynonymous SNVs detected at the RNA level in breast tumors and healthy breast tissues derived from reconstruction surgery were employed to define a variant-specific library against which the DIA data was searched. Panel (D) shows in which samples (healthy breast tissue and cancer) each variant was detected (Numbers in brackets represent peptide charge). Abbreviations: DIA: data-independent acquisition, DTU: differential transcript usage, MS: mass spectrometry, SAAV: single amino acid variant, SNV: single nucleotide variant.

Out of the 539 differentially used transcripts in the ER+/– comparison, 127 were detectable at the proteomic level (DIA level peptide FDR < 0.01). Of these, 6 were identified by isoform-specific peptides. Out of 63 identified DTUs between appearance-define groups, only one had a matching peptide (Figure 5A). Here, we observed that peptide-level DTUs could recapture significant differences observed at the transcript level (Figure 5B,C), such as PTCD3 (*p*-value = 0.018). In this regard, we assume that the detected discrepancies between transcript and isoform-specific peptide abundances might either relate to post-translational regulation of protein abundance, as shown in recent studies.^12^ The differential expression of variant-specific transcript/proteins between ER-positive and ER-negative patients suggests a different functional role for isoforms of the same protein.

A similar approach was applied to test the detection of SAAVs (see Experimental Procedures and Figure S1D). So far, there has only been a limited number of SAAVs that have been confirmed using protein measurement techniques, as antibody-based techniques typically are unable to distinguish SAAVs, and MS-based proteomics experiments typically suffer from the limited coverage of peptides per protein. In our workflow, we can partly circumvent this problem by specifically targeting peptides with known SAAVs, resulting in the identification of nine high-confident peptides with SAAVs (Figure 5D). Annotation of the quantified SAAV peptides revealed that several of the peptides that were identified in multiple samples stemmed from variants known to be prevalent in the Nordic population. One SAAV-specific peptide however (ATP5PF V69I, Figure 5D) was detectable in only one tumor and had no documented prevalence in the Nordic population, indicating a case of a novel protein-level identification of a cancer mutation or rare germline mutation. Altogether, these results serve as proof of the detectability of splicing and mutational events using our proposed workflow.

### Molecular Signature Evaluation and Drug-Targeting Strategies Based on Integrated Data

In the final analysis, we assessed whether protein–transcripts pairs showed similar trends for key gene signatures currently being explored in routine diagnostic analyses (Mammaprint, Oncotype-DX, and PAM50^35^) and targets of FDA-approved drugs. For the established prognostic signatures such as MammaPrint, Oncotype-DX, and the PAM50 classifier, the protein–transcript pairs generally displayed higher than median-correlation coefficients (Figure S11A,B), as also shown by a recent study.^12^ These results suggest a high robustness of these markers and the partial transferability of prognostic signatures from transcriptomics to proteomics. In contrast to the prognostic signatures, FDA drug targets displayed a considerably wider correlation range (Spearman Rho range: −0.475 to 0.740). For this, we investigated the discrepancies displayed in this subset further.

Upon evaluating the overall transcript–protein correlation distributions, we observed poorly correlating transcript protein pairs enriched for specific pathways (Figure 2). Given the fact that transcript/protein expression and regulation is also dependent on upstream regulators of cell biology (e.g., transcription factors), we argued whether Food and Drug Administration (FDA) drug target transcript–protein correlations were dependent on the ER status.

**Figure 6:**
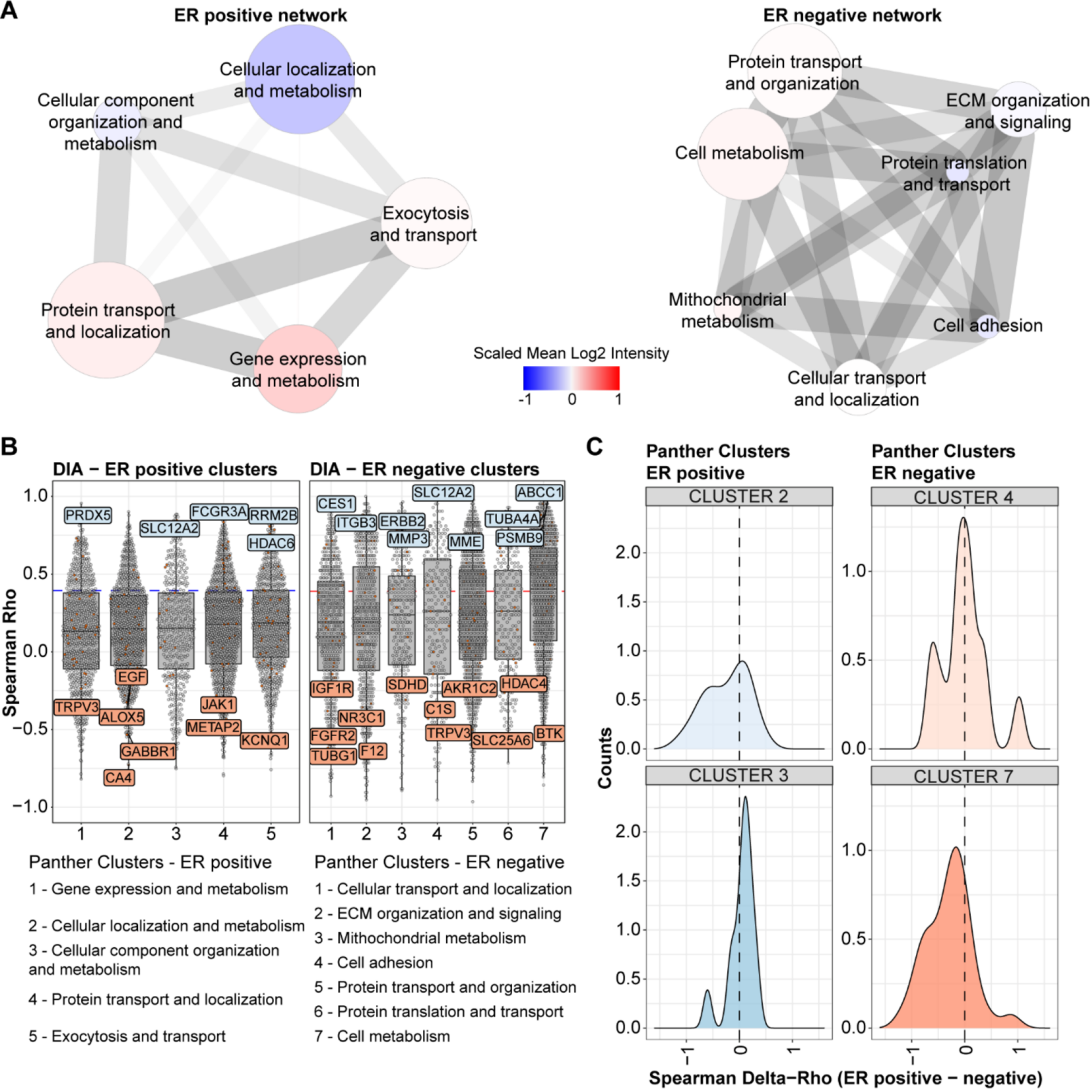
Protein cluster regulation dependent on the estrogen receptor status. Co-regulated protein clusters in ER-positive (left) and ER-negative (right) tumors (see Figure S15) were extracted from the DIA data, annotated with GOBP terms, condensed, and visualized in Cytoscape (A). Edge thickness and length relate to the cluster distance (Euclidean), the node color relates to the scaled mean intensity of all proteins in each cluster, and the node size depends on the number of proteins in each cluster. Panel (B) shows the correlation to mRNA of each protein per cluster for ER-positive and ER-negative tumors. Panel (C) displays differences in correlation to RNA between ER-positive and ER-negative (i.e., ER positive–ER negative) tumor groups within showcased co-regulation clusters for FDA drug targets. Abbreviations: DIA: data-independent acquisition, ER: estrogen receptor, FDA: Food and Drug Administration, GOBP: gene ontology biological process, MS: mass spectrometry.

Evaluation of transcript–protein correlations by the tumor subgroup revealed that ∼25% of the transcript–protein pairs often displayed radically different correlations dependent on the ER status (e.g., PARP1, Figure S12A) or tumor appearance (e.g., MMP2, Figure S12B). Within ER-positive and ER-negative tumors, pairs displaying disagreeing correlations generally belonged to metabolism, protein localization, and cellular transport networks (Figures S13 and S14). To further clarify this (the list of all proteins correlations is reported in Tables S10 and S11) and assess whether different correlation between ER-positive and ER-negative tumors were associated to specific protein networks, we extracted protein co-regulation clusters (i.e., groups of highly correlated proteins) from the ER-positive and ER-negative subsets of our proteomic layers (Figures S15A and S16A). We selected a minimum number of clusters using the elbow method (see Experimental Procedures) for each tumor group (Figures S15B and S16B), and the most enriched gene ontology terms were used to annotate each cluster. The annotation distance between each cluster was calculated (Figures S15C,D and S16C,D), and the number of clusters was then condensed using a second iteration of the elbow method (Figures S15E and S16E). The so-derived clusters comprised metabolism, cellular transport, and immune response (Figure 6A and Figure S17A). Here, co-regulated protein clusters showed different correlations with RNA data in relation to the ER status (e.g., cell metabolism, Figure 6B and Figure S17B). Furthermore, FDA drug targets displayed shifts in RNA–protein correlation coefficients within several co-regulated clusters (e.g., the cell adhesion cluster in ER-negative clusters out of DIA MS data Figure 6C; cell secretion and immune signaling in ER-negative clusters, Figures S17C and S18). These data suggest that subsets, or entire clusters of protein co-regulation networks, display different degrees of agreement between RNA and protein data dependent on the tumor subgroup. This results in groups of proteins that, e.g., under regulation of the ER, display better correlations with RNA than the same proteins expressed in ER-negative tumors, indicating that their regulation is impacted by ER expression.

Despite the fact that diverging correlations were not significant, likely in relation to the small number of samples included here, these results may open new grounds for investigation of gene/protein regulation in tumor groups/subtypes. For this, further validation is necessary to confirm these results, where potential prognostic markers should be evaluated taking into account key tumor subgroups/features and the extent of protein post-translational regulation or class.

Collectively, these results indicate that differentially regulated protein networks exist in clinically relevant sample groups and that these protein networks affect the abundance of potential biomarkers and drug targets. On the one hand, we conclude that the evaluation of new biomarkers can be restricted to the nucleic acid or protein level analysis. On the other hand, drug treatments affect proteins in cells, for which a protein-wide evaluation of these targets is of considerable relevance. Furthermore, integrated studies followed by functional assays should be the method of choice to shed light on the fine regulation of key cancer genes and their protein products.

## Discussion

BC is the most common malignancy in women, although its death rate continues to decline due to constant advancements in clinical care, drug target development, and better definition of tumor biology.^1^ Several key mechanisms underlying breast cancer biology have been elucidated in detail over the years, such as the immunogenicity of triple negative tumors and the action of the ER transcription factor in ER-positive cancers.^36^ Despite this, the mechanisms underlying breast cancer therapy resistance (e.g., *ESR1* mutations^37^), or other prognostic factors such as the mammographic appearance,^6^ have yet to be thoroughly investigated.

In BC, recent studies have shown that the integration of genomic and proteomic approaches expanded the knowledge in biological networks underlined by the intrinsic molecular subtypes and suggested that discrepancies in abundance between RNA and protein data might derive from RNA and/or protein regulation mechanisms.^11,12^

Most proteogenomic studies employ massive sample fractionation and DDA MS acquisition methods to achieve high proteome coverage, resulting in an extensive measurement time. Here, we improved novel computational workflows to improve the capabilities of DIA MS in proteogenomic studies. By employing naturally occurring retention time peptides rather than spiked-in internal retention time (nRT) ones, our workflow achieved improved peptide identification accuracy and high identification rates.

We here employed DIA MS to demonstrate the high proteome depth and quantitative accuracy out of single-shot analyses, compare transcriptome data to protein analyses, and assess the capability of DIA in detecting genomic features such as DTU and SAAVs and to investigate biological pathways underlying understudied tumor features. The combined output from the workflows improved the identification rates of DIA MS. The spectral library used for DIA MS data analysis was based on DDA MS runs for 21 breast cancer tissues and 4 normal breast specimens, which was further extended with RNA-guided assays for SAAVs and DTU. The number of identifications we obtained through DIA MS was similar to the ones achieved through fractionated DDA analysis of the same samples though with fewer missing observations. A second set of 24 samples was then employed to validate our findings out of RNA–protein correlation and spiculated morphology enrichment analyses.

Targeted analysis of changes in the mutational and splicing landscapes using informed spectral assays enabled quantification of SAAV- and DTU-specific peptides. Although the number of quantified SAAVs and DTUs was relatively sparse, the possibility of using genomic-informed assumptions to identify the translation of splicing and mutational events at the protein level opens up new possibilities for future studies. In particular, evaluation of DTU-specific peptides is of importance due to their repercussions on protein function or activity, with downstream effects on molecular dynamics and on the evaluation of patient outcome.^38^ To better define the role of the aforementioned features at the protein level, further improvements to the computational workflows and data acquisition strategies are required to yield a higher number of identifications out of our DIA data, which can be recurrently mined.

Upon comparing the dynamic range of our transcriptomic and proteomic datasets, we noticed that most transcript–protein pairs displayed similar quantitative levels across our dataset, as observed in a previous study.^39^ Only a small subset of proteins displayed negative correlation coefficients with their matching transcript abundances. While these results might relate to the small sample number in our cohorts, repeated observation of these findings across sample sets 1 and 2 increased the confidence in our results. In addition to this, these findings confirm observations from previous reports, where mRNA and protein abundances either display positive significant correlations or decoupled measurements (i.e., nonsignificant correlations; gray bars in Figure 2B and Figure S6A), with the latter due to RNA-independent regulation at the protein level like post-translational modification, ubiquitination, etc.^12^ Here, we discovered that proteins involved in processes such as splicing and translational regulation tend to correlate poorly with their transcripts, as opposed to those belonging to immune-related pathways. In this case, cellular processes controlling cellular turnover such as ubiquitination and proteasomal degradation,^40^ miRNA activity,^41,42^ or epigenetic factors may actually be responsible for these quantitative discrepancies and target-specific protein clusters. The relatively low number of negatively correlated protein–transcript pairs suggests that post-translational regulation might indeed target-specific protein groups but the impact on the entire proteome might not be as extensive as previously thought.^43^ For this, further studies of functional nature are required to verify such claims.

Following our analysis of enriched gene-protein pairs and pathways expressed according to the expression of key transcription factors (ER status) or tumor appearances (spiculation), we noticed discrepancies between transcript and protein levels of a subset of differentially expressed genes. While these transcript–protein pairs did not enrich for pathways previously associated to rapid protein turnover and consequential poor RNA–protein correlation, we cannot exclude the action of such molecular mechanisms. In fact, this discrepant subset might have been too small to enable us to see any significant association to protein regulation. Interestingly, RNA and MS measurements converged at the pathway level, as also shown in a previous study.^11^ This was especially true for previously characterized breast cancer pathways, such as the enrichment of ER responsive genes or immune signaling molecules in ER-positive and ER-negative tumors, respectively. In addition to this, our analyses elucidated relevant molecular differences between spiculated and nonspiculated appearances, where tissue remodeling and EMT pathways were found enriched in the former and inflammation- and proliferation-related networks were enriched in the latter. These results confirm that breast cancer invasion of the surrounding tissue through spiculation has been generally associated with stromal and extracellular matrix remodeling. The results also imply that a possible different transcriptional program takes place in these cancers, though further experimental verification is needed. While the EMT pathway has been reasoned to be a mutable transcriptional program,^44^ with cells acquiring a spectrum of biological features related to epithelial or mesenchymal fates, our data indicates that invasion of normal tissues through spiculae might rely on a mesenchymal cancer cell front. In the light of this, future studies are necessary to confirm these results through, for example, mechanistic experiments such as overexpression studies. These would aim to clarify the molecular mechanisms related to EMT activation and to establish the association between these features and patient prognosis.

Based on the results that transcriptomic and proteomic analyses largely converge at the pathway level, we further investigated if this also holds true for biomarkers or drug targets. Interestingly, we observe that transcript–protein pairs belonging to established predictive signatures (e.g., Mammaprint) display a high level of correlation, thus suggesting the transferability of these biomarker panels onto the proteomic level. In contrast, this did not hold true for FDA-approved drug targets. Since a previous study has shown that post-translational regulation mechanisms might significantly impact protein abundances of drug targets,^12^ we hypothesized that such mechanisms might be operating at different activity levels within critical subgroups such as ER-positive and ER-negative tumors. Despite the fact that we were able to only partially validate the dependency of genes displaying diverging RNA-protein correlations between critical tumor groups (i.e., ER positive/negative), we believe that different protein regulation mechanisms operate within these subgroups. Further experiments to validate this hypothesis are needed.

Overall, FDA-approved drug targets displayed variable degrees of concordance between the two data layers, with foreseeable repercussions in biomarker identification and monitoring dependent on the measurement technology as well as tumor subgroup inherent biology. The expression of differential or mutually exclusive transcriptional programs or regulatory mechanisms is a known factor in cancer.^45^ Transcriptional programs impact tumor diversity, establishing cellular changes through genetic and epigenetic mechanisms.^46,47^ These mechanisms may indeed affect genes and proteins on different levels to alter their expression. As an example, ER-positive and ER-negative tumors proliferate via the activation of different proliferation signaling pathways (e.g., ER signaling *vs* MYC), which in turn are under different regulation. This might be related to the discrepancies in the expression of RNA–protein pairs that we observed in our sample set.

For this, we find it to be imperative to overlay genomic and proteomic information to (i) determine disease subgroups with altered gene and protein expression clusters, (ii) use such information to derive tumor proteotype-specific biomarkers or alternative drug targets, and (iii) choose the most appropriate treatment strategy based on the tumor subgroup. These findings indicate that the evaluation of protein levels should be performed for a subset of the proteome when evaluating the association of potential markers in the clinical laboratory or when using mRNA as a substitute for protein abundance. These results support the complementarity of genomic and proteomic information in the dissection molecular pathologies, such as the definition of pathways of interest for further functional assessment and/or drug testing. It is important, however, to point out that this study is based on a relatively small patient sample set, which limits the generalization of significant findings out of our analyses. This is especially relevant when considering the discrepancies of RNA–protein abundance (e.g., negative correlation), where a significantly bigger dataset would have allowed better elucidation of such findings.

In conclusion, we have here established and benchmarked an improved DIA MS-based workflow in proteogenomic studies to identify mutational processes at the protein level and the discrepancies that arise between mRNA and protein quantitative data layers, which are in turn dependent on transcript and protein regulation processes. Our analyses also validated previously established enrichments of estrogen receptor-dependent molecular features associated to transcription factor expression and provided evidence of molecular differences related to the development of mammographic morphologies in spiculated tumor masses. These results suggest that there are differentially regulated protein networks in clinically relevant sample groups and that these protein networks impact both cancer biology and the abundance of potential biomarkers and drug target abundance. Validation of such claims via large-scale studies is needed.

In addition to this, to assess whether these findings related to biological regulation of protein stability or mRNA translation rates, biochemical and genetic/epigenetic studies should be performed by for example functional high-throughput knockdown models.

In conclusion, the data presented here establish a new DIA-based proteogenomic workflow for the analysis of clinical specimens. While our results shed light on the biological processes related to tumor altered morphology, deeper evaluation of the proteogenomic features presented here is needed. This will enable not only a better understanding of breast tumor biology but also the development of new therapies or biomarkers.

## Supporting Information Available

(Table S1) Clinical characteristics of breast cancer cohorts, (Figure S1) computational workflow generated to analyze the DIA dataset, (Figure S2) scheme of DIA search and overview of separate library search results, (Figure S3) impact of the sample preparation method on proteomic identifications, (Figure S4) sample-level peptide and protein identifications within proteomic layers, (Figure S5) evaluation of *p*-value distribution out of RNA-protein correlation analyses, (Figure S6) correlation distribution between RNA and proteomic data in the validation dataset, (Figure S7) correlation of RNA-protein correlations between DDA and DIA data layers, (Figure S8) significant transcript–protein pairs pathway-wise annotation and correlations, (Figure S9) analysis of immunohistochemical stainings, (Figure S10) confirmation of integrated findings in the validation dataset, (Figure S11) evaluation of biomarker signatures, (Figure S12) RNA–protein correlations based on tumor subgroups, (Figure S13) pathway representation of transcript–protein pairs displaying discordant correlations between ER-positive and ER-negative tumors, (Figure S14) pathway representation of transcript–protein pairs displaying discordant correlations between tumor appearance groups, (Figure S15) co-regulation clusters in ER-positive and ER-negative tumor samples out of the DDA data layer, (Figure S16) co-regulation clusters in ER-positive and ER-negative tumor samples out of the DIA data layer, (Figure S17) protein cluster regulation dependent on the estrogen receptor status out of the DDA data layer, and (Figure S18) difference in correlation coefficients in clusters of regulation dependent on the estrogen receptor status out of the DIA data layer (PDF)
(Table S2) Clinical and histopathological information per sample (XLSX)
(Table S3) Cata-independent acquisition settings (XLSX)
(Table S4) Sample set 1: proteomic data (DDA layer) (XLSX)
(Table S5) Sample set 1: proteomic data (DIA layer) (XLSX)
(Table S6) Sample set 2: proteomic data (DIA layer) (XLSX)
(Table S7) Correlation between RNA and protein (sample set 1 correlations; RNA-DDA and RNA-DIA) (XLSX)
(Table S8) Significant genes out of ER-status analysis of the RNA-sequencing dataset matching MS data (ER-status DESEQ2 output matched against protein lists) (XLSX)
(Table S9) Significant genes out of spiculation-status analysis of the RNA-sequencing dataset matching MS data (appearance-status DESEQ2 output matched against protein lists) (XLSX)
(Table S10) Correlations between RNA and protein data by the ER status (sample set 1 correlations within ER-positive and ER-negative tumors, RNA-DDA and RNA-DIA) (XLSX)
(Table S11) Correlations between RNA and protein data by appearance (sample set 1 correlations within spiculated and nonspiculated tumors, RNA-DDA and RNA-DIA) (XLSX)

## Notes

Transcriptomic data (samples sets 1 and 2) was uploaded to Mendeley Data under DOI: 10.17632/wmkb7z7mz4.1. Raw sequence data was not uploaded in accordance with Swedish law, which prevents the upload of person-specific and person-identifying information. For the initial set of 21 breast cancer samples (sample set 1), DDA and DIA MS data, and their respective search result files have been deposited to the ProteomeXchange Consortium *via* the PRIDE partner repository^48^ with the dataset identifier: PXD018830. The RAW and search result files for the 24-sample set (sample set 2) are accessible under identifier: PXD021394.

## Abbreviations

AGC: automatic gain control
BANDITs: Bayesian analysis of differential splicing
BC: breast cancer
BCA: bicinchoninic acid assay
DDA: data-dependent acquisition
DIA: data-independent acquisition
DTU: differential transcript usage
EMT: epithelial-to-mesenchymal transition
ER: estrogen receptor
FDA: Food and Drug Administration
FDR: false discovery rate
FFPE: formalin-fixed and paraffin-embedded
FT: flow-through
FA: formic acid
GSEA: gene set enrichment analysis
GOBP: gene ontology biological process
GTL: generic transition list
HCD: higher energy induced collision dissociation
LFQ: label-free quantification
PgR: progesterone receptor
PSM: peptide spectral match
RIPA: radioimmunoprecipitation assay
RT: retention time
SAAV: single amino acid variant
SAX: strong anion exchange
SNV: single nucleotide variant
SWATH: sequential window acquisition of all theoretical mass spectra
WTL: whole tissue lysate
