# Supplementary material for: Proteogenomic Workflow Reveals Molecular Phenotypes Related to Breast Cancer Mammographic Appearance
Source: J Proteome Res. 2021 Apr 15;20(5):2983–3001. doi: 10.1021/acs.jproteome.1c00243 (PMC8155562; doi:10.1021/acs.jproteome.1c00243)
Supplement: Supplementary file 1 — pr1c00243_si_001.pdf [file pr1c00243_si_001.pdf]

SUPPLEMENTARY INFORMATION

TITLE: "Proteogenomic workflow reveals molecular phenotypes related to breast cancer mammographic appearance"

Authors

Tommaso De Marchi[1,11,*], Paul Theodor Pyl[1,9,11], Martin Sjöstrom[1,10], Stina Klasson[2], Hanna Sartor[3], Lena Tran[1], Gyula Pekar[4], Johan Malmström[5], Lars Malmström[6-7], Emma Niméus[1,8,*]

Author affiliations

1. Division of Surgery, Oncology, and Pathology, Department of Clinical Sciences, Lund University, Solvegatan 19, SE-223 62, Lund, Sweden.
2. Department Plastic and Reconstructive Surgery, Skåne University Hospital, Inga Marie Nilssons gata 47, SE-20502, Malmö, Sweden.
3. Division of Diagnostic Radiology, Department of Translational Medicine, Skåne University Hospital, Entrégatan 7, SE-22185, Lund, Sweden.
4. Division of Oncology and Pathology, Department of Clinical Sciences, Lund University, Skåne University Hospital, SE-22185, Lund, Sweden.
5. Division of Infection Medicine, Department of Clinical Sciences Lund, Faculty of Medicine, Lund University, Klinikgatan 32, SE-22184, Lund, Sweden.
6. S3IT, University of Zurich, Winterthurerstrasse 190, CH-8057, Zurich, Switzerland.
7. Institute for Computational Science, University of Zurich, Winterthurerstrasse 190, CH-8057, Zurich, Switzerland.
8. Department of Surgery, Skåne University Hospital, Lund, Sweden.
9. Current affiliation: Department of Laboratory Medicine, National Bioinformatics Infrastructure Sweden, Science for Life Laboratory, Kansli ILM, BMC, I:12, Sölvegatan 19, SE-221 00 Lund, Sweden.
10. Current address: Department of Radiation Oncology, University of California, San Francisco, 1450 3rd Street, CA 94158, San Francisco, USA.
11. These authors equally contributed to this study.

*Corresponding authors.

Supporting Information – Table of Contents

Supplemental Tables

- Table S1. Clinical characteristics of breast cancer cohorts.
- Table S2. Clinical and histopathological information per sample. (XLSX)
- Table S3. Data Independent Acquisition settings. (XLSX)
- Table S4. Sample Set 1 - Proteomic data (DDA-layer). (XLSX)
- Table S5. Sample Set 1 - Proteomic data (DIA layer). (XLSX)
- Table S6. Sample Set 2 - Proteomic data (DIA layer). (XLSX)
- Table S7. Correlation between RNA and protein (Sample Set 1 correlations; RNA-DDA and RNA-DIA). (XLSX)
- Table S8. Significant genes out of ER status analysis of the RNA-sequencing dataset matching MS data (ER-status DESEQ2 output matched against protein lists). (XLSX)
- Table S9. Significant genes out of spiculation status analysis of the RNA-sequencing dataset matching MS data (Appearance-status DESEQ2 output matched against protein lists). (XLSX)
- Table S10. Correlations between RNA and protein data by ER status (Sample Set 1 correlations within ER positive and ER negative tumors; RNA-DDA and RNA-DIA). (XLSX)
- Table S11. Correlations between RNA and protein data by Appearance (Sample Set 1 correlations within spiculated and non-spiculated tumors; RNA-DDA and RNA-DIA). (XLSX)

Supplemental Figures

- Figure S1. Computational workflow generated to analyze the DIA dataset (schematic overview of DIA computational workflow).
- Figure S2. Scheme of DIA search and overview of separate library search results (DIA-search workflow and summary of identifications).
- Figure S3. Impact of sample preparation method on proteomic identifications (Library peptide/protein identification contribution dependent on sample preparation).
- Figure S4. Sample-level peptide and protein identifications within proteomic layers.
- Figure S5. Evaluation of p-value distribution out of RNA-protein correlation analyses (RNA-protein analyses in Sample Set 1).
- Figure S6. Correlation distribution between RNA and proteomic data in the validation dataset (RNA-protein analyses in Sample Set 2).
- Figure S7. Correlation of RNA-protein correlations between DDA and DIA data layers (Sample-wise RNA-DDA vs RNA-DIA correlations).
- Figure S8. Significant transcript-protein pairs pathway-wise annotation and correlations (Correlation of RNA-proteins across significantly enriched pathways for ER status analsys).
- Figure S9. Analysis of immunohistochemical stainings (Histopathological and immunohistochemical evaluation of tissues out of Sample Set 1).
- Figure S10. Confirmation of integrated findings in the validation dataset (RNA-protein comparison at the level of differential expression and pathway enrichment for ER status and appearance).
- Figure S11. Evaluation of biomarker signatures (RNA-protein correlation across biomarker signatures and FDA drug targets).

- Figure S12. RNA-protein correlations based on tumor subgroup (RNA-protein correlation within ER status and appearance tumors).
- Figure S13. Pathway representation of transcript-protein pairs displaying discordant correlations between ER positive and negative tumors.
- Figure S14. Pathway representation of transcript-protein pairs displaying discordant correlations between tumor appearance groups.
- Figure S15. Co-regulation clusters in ER positive and negative tumor samples out of the DDA data layer (Summary of step-wise definition of protein co-regulation groups across ER positive and negative tumors at the DDA level).
- Figure S16. Co-regulation clusters in ER positive and negative tumor samples out of the DIA data layer (Summary of step-wise definition of protein co-regulation groups across ER positive and negative tumors at the DIA level).
- Figure S17. Protein cluster regulation dependent on Estrogen Receptor status out of the DDA data layer (Evaluation of co-regulation cluster dependency of RNA-protein correlations, DDA data layer).
- Figure S18. Difference in correlation coefficients in clusters of regulation dependent on Estrogen Receptor status out of the DIA data layer (Extended view of cluster co-regulation, Fig 6C, DIA data layer).

Supplementary Table

Table S1. Clinical characteristics of breast cancer cohorts.

	**Breast cancer tissues (Sample Set 1; n = 21)**		**Breast cancer tissues (Sample Set 2; n = 24)**	
	**N**	%	N	%
**Age***				
**≤ 55 years**	8	38.1	14	58.3
**> 55 years**	11	52.4	10	41.7
**Tumor size**				
**T1 (≤ 2cm)**	14	66.7	13	54.2
**T2 (2-5cm) + Tx**	7	33.3	11	45.8
**ER**				
**Negative**	8	38.1	7	29.2
**Positive**	13	61.9	17	70.8
**PgR***				
**Negative**	8	38.1	11	45.8
**Positive**	13	61.9	11	45.8
**Appearance**				
**Spiculated**	7	33.3	4	16.7
**Non-spiculated**	14	66.7	20	83.3

*Patients with missing characteristics are not reported

Abbreviations: ER, Estrogen Receptor; PgR, Progesterone Receptor.

Supplementary Figures

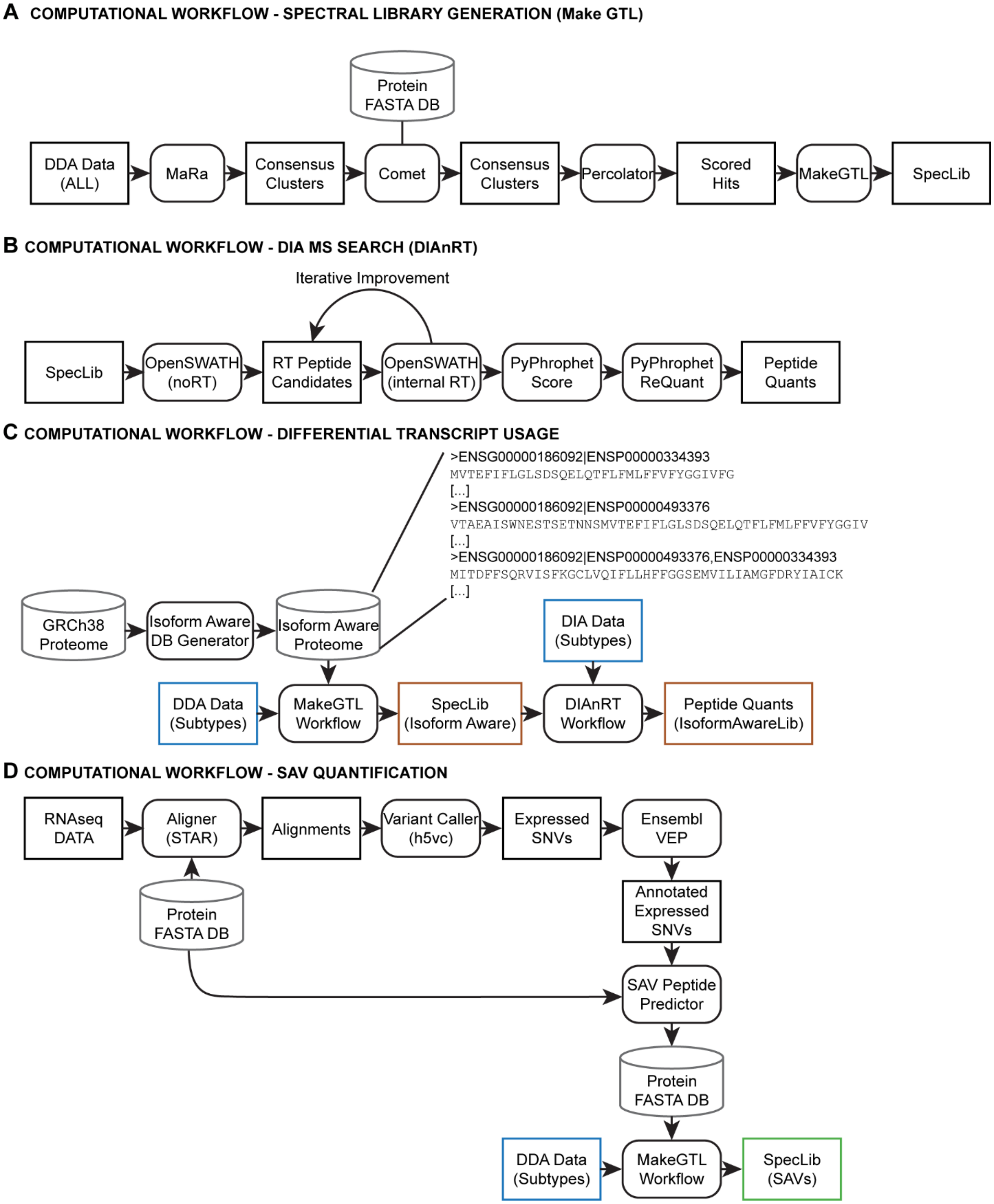

Figure S1. Computational workflow generated to analyze the DIA dataset.

Computational workflows (see Methods for details) were established in this study to generate spectral libraries (A) for DIA data search (B), assess differential transcript usage at the proteomic level (C), and detect and quantify SAVs (D).

Abbreviations: DB: Database; DDA: Data Dependent Acquisition; DIA: Data Independent Acquisition; RT: Retention Time; SAV: Single Aminoacid Variant; SNV: Single Nucleotide Variant; VEP: variant Effect Predictor.

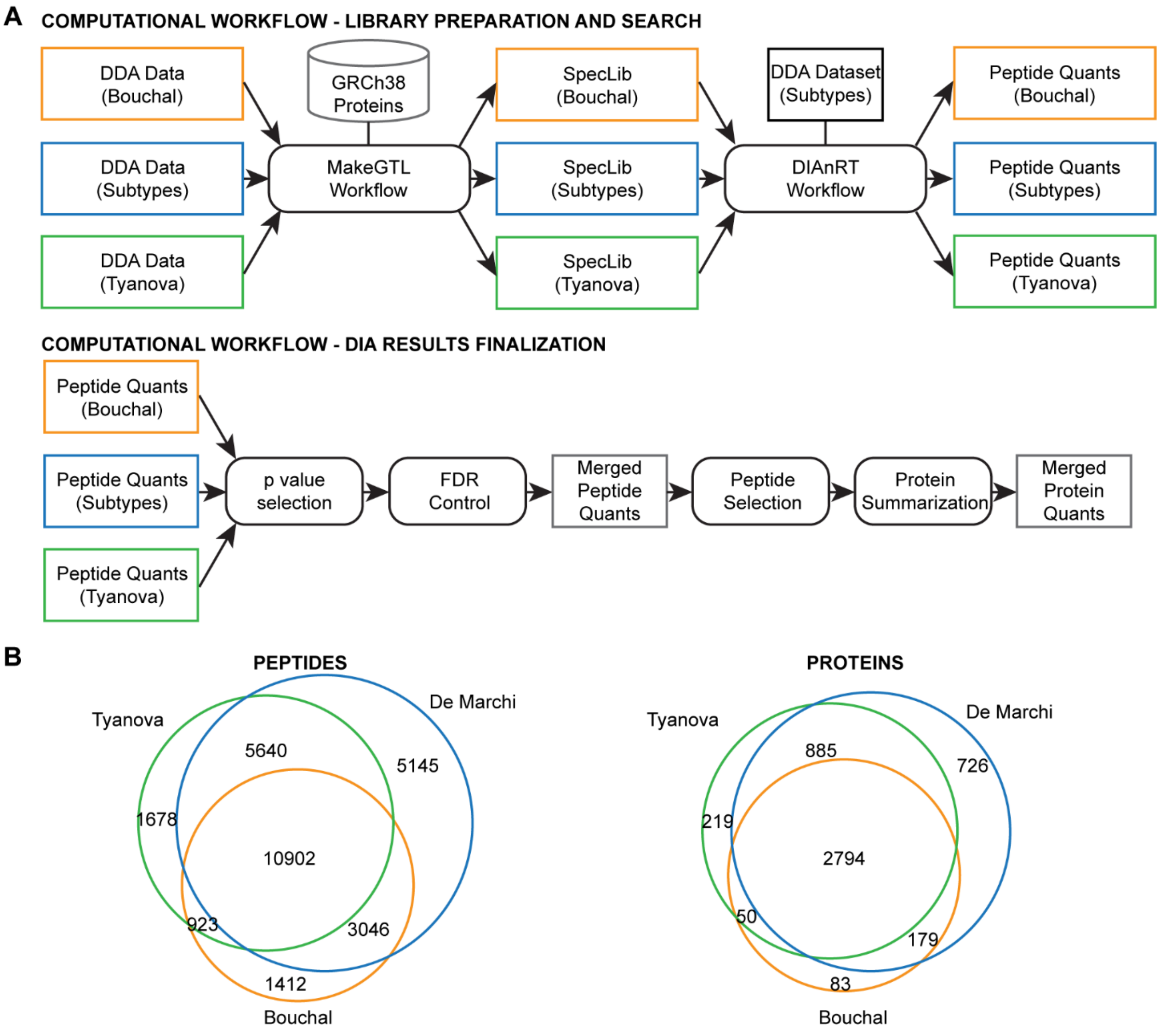

Figure S2. Scheme of DIA search and overview of separate library search results.

Panel A displays a schematic overview of DIA library preparation for all three datasets (i.e. this study (De Marchi), Bouchal and Tyanova; upper panel) and finalization of search results (lower panel). Panel B displays identification overlap for peptides (left) and proteins (right) across the three libraries.

Abbreviations: DIA: Data Independent Acquisition.

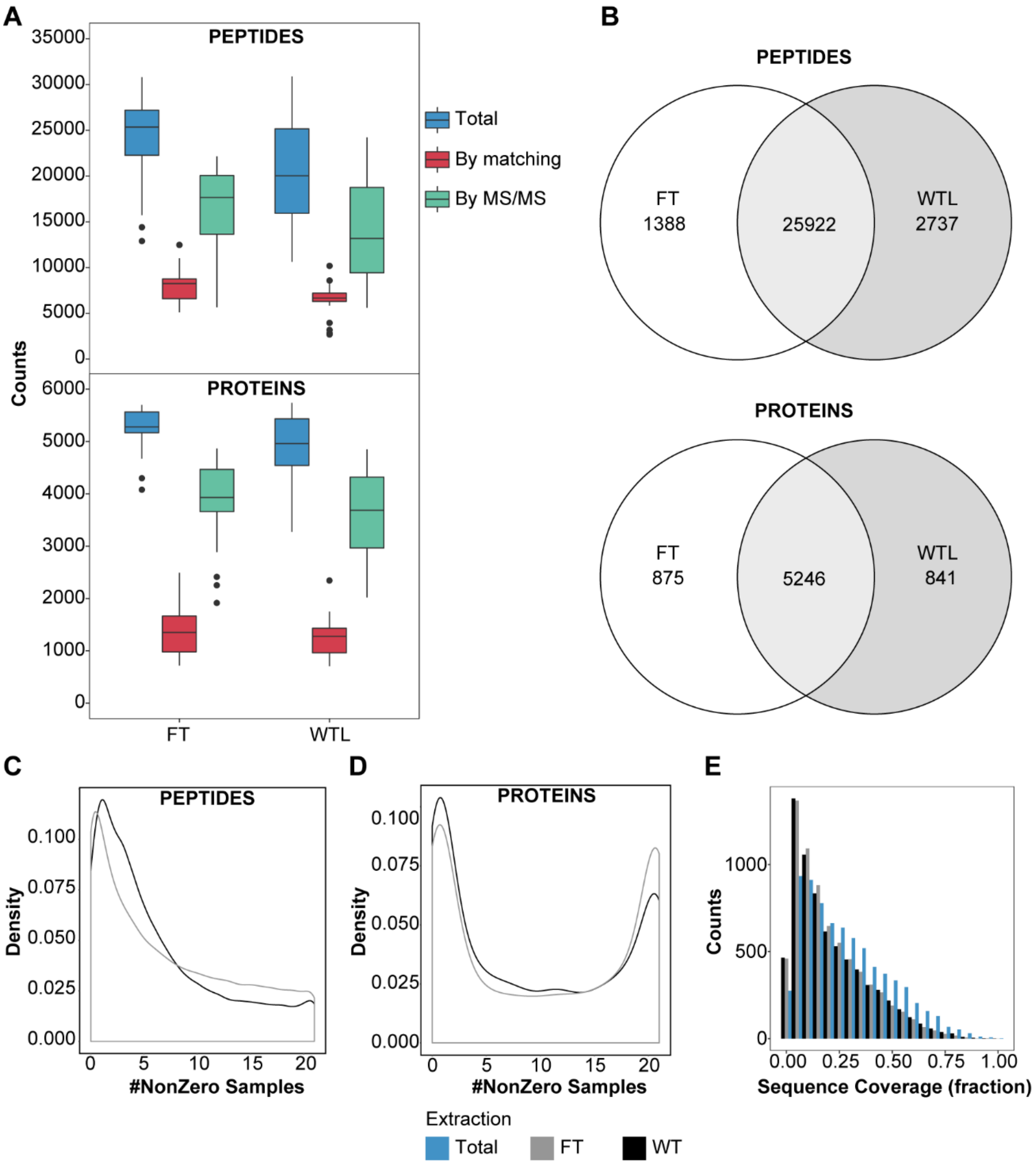

Figure S3. Impact of sample preparation method on proteomic identifications.

Samples for DDA MS-based library generation were prepared as ALLPREP protein flowthroughs (FT) and whole tissue lysates (WTL). After MaxQuant search, we compared the number of unique peptides and proteins identified in each subgroup (A) and their overlap (B). Data density for peptides (C) and proteins (D) were then compared and found to be highly similar. At the level of sequence coverage (E) the two subsets were highly comparable, though their combination showed a slightly better performance.

Abbreviations: DDA: Data Dependent Acquisition; FT: Flowthrough; MS: Mass Spectrometry; WTL: Whole Tissue Lysate.

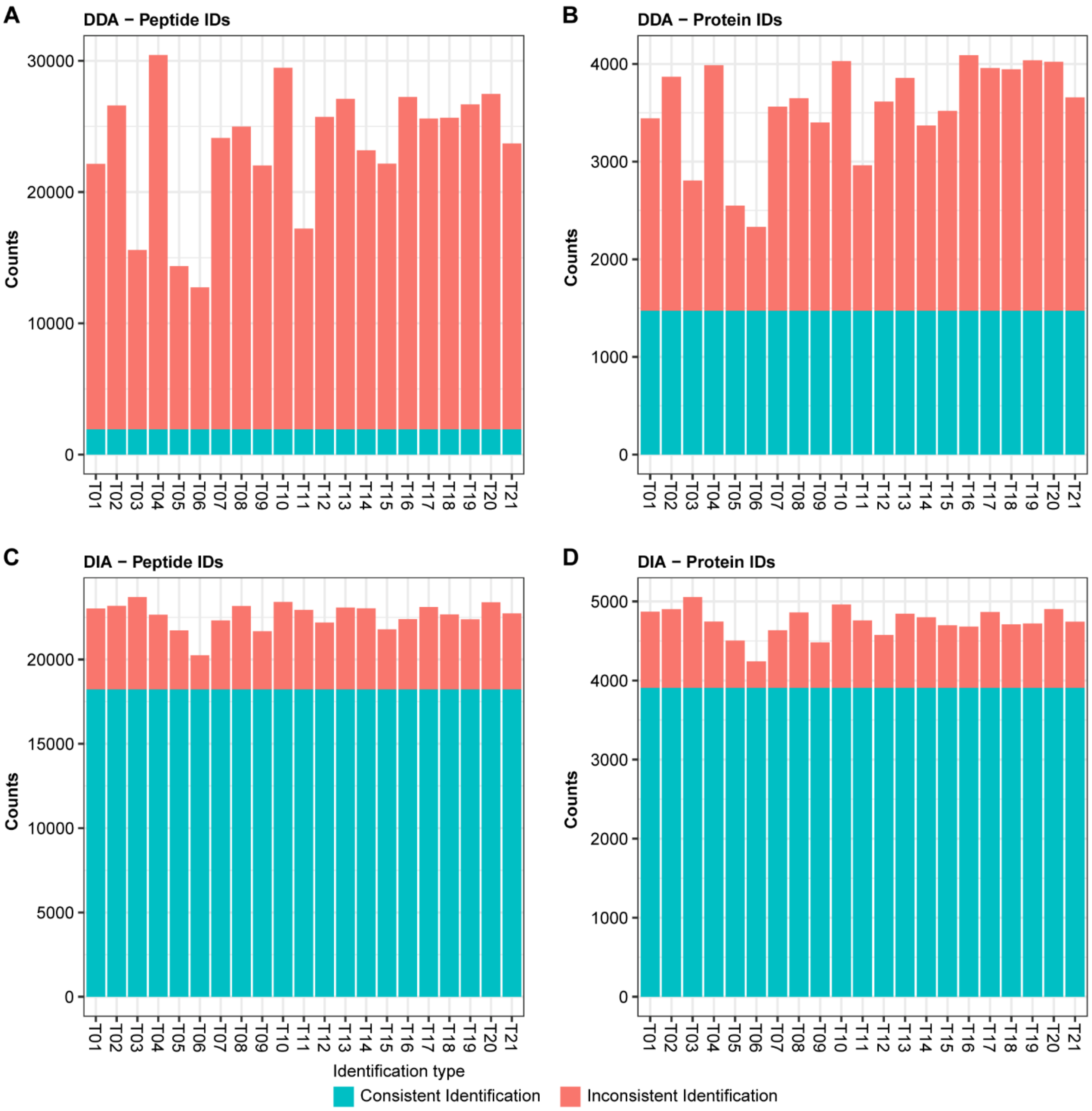

Figure S4. Sample-level peptide and protein identifications within proteomic layers.

Sample-wise DDA and DIA peptide and protein identifications. Panel A and B display results peptide and protein identifications for DDA, respectively. Panel C and D display results peptide and protein identifications for DIA, respectively. Common identifications through the sample set are shown in cyan, while non-consistent ones are displayed in light red.

Abbreviations: DDA: Data Dependent Acquisition; DIA: Data Independent Acquisition.

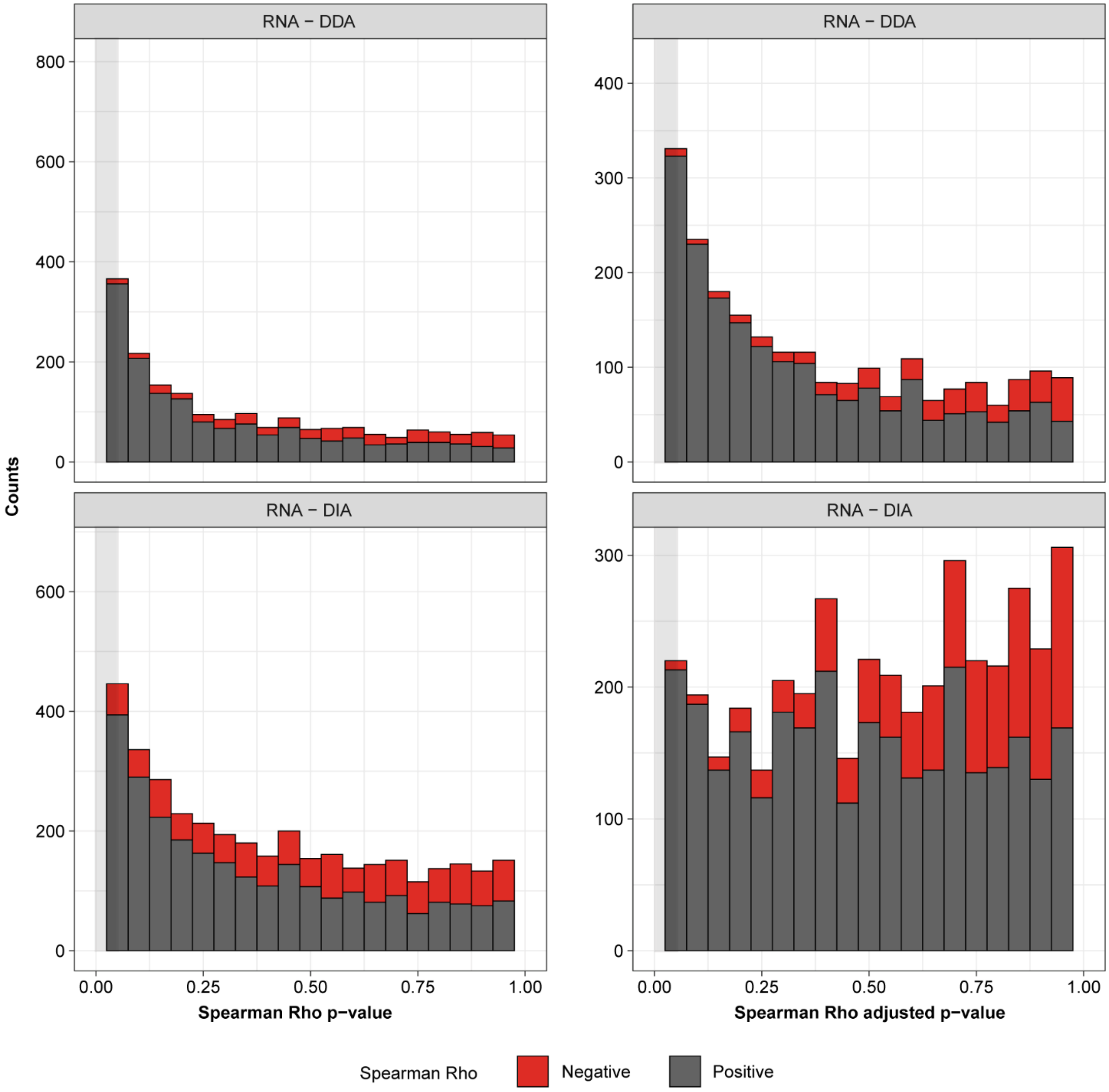

Figure S5. Evaluation of p-value distribution out of RNA-protein correlation analyses.

Following our correlation analyses between matching transcripts and proteins (reported in Fig. 2), we evaluated the distribution of the negative and positive correlation coefficients-related p-values. We noticed that the uncorrected p-values from negative Spearman correlations seemed to follow a background-like distribution (left panels). These p-values became non-significant after Benjamini-Hochberg correction (right panels), thus suggesting that they might not be statistically meaningful or that the effect size might be too small.

Abbreviations:DDA: Data Dependent Acquisition; DIA: Data Independent Acquisition.

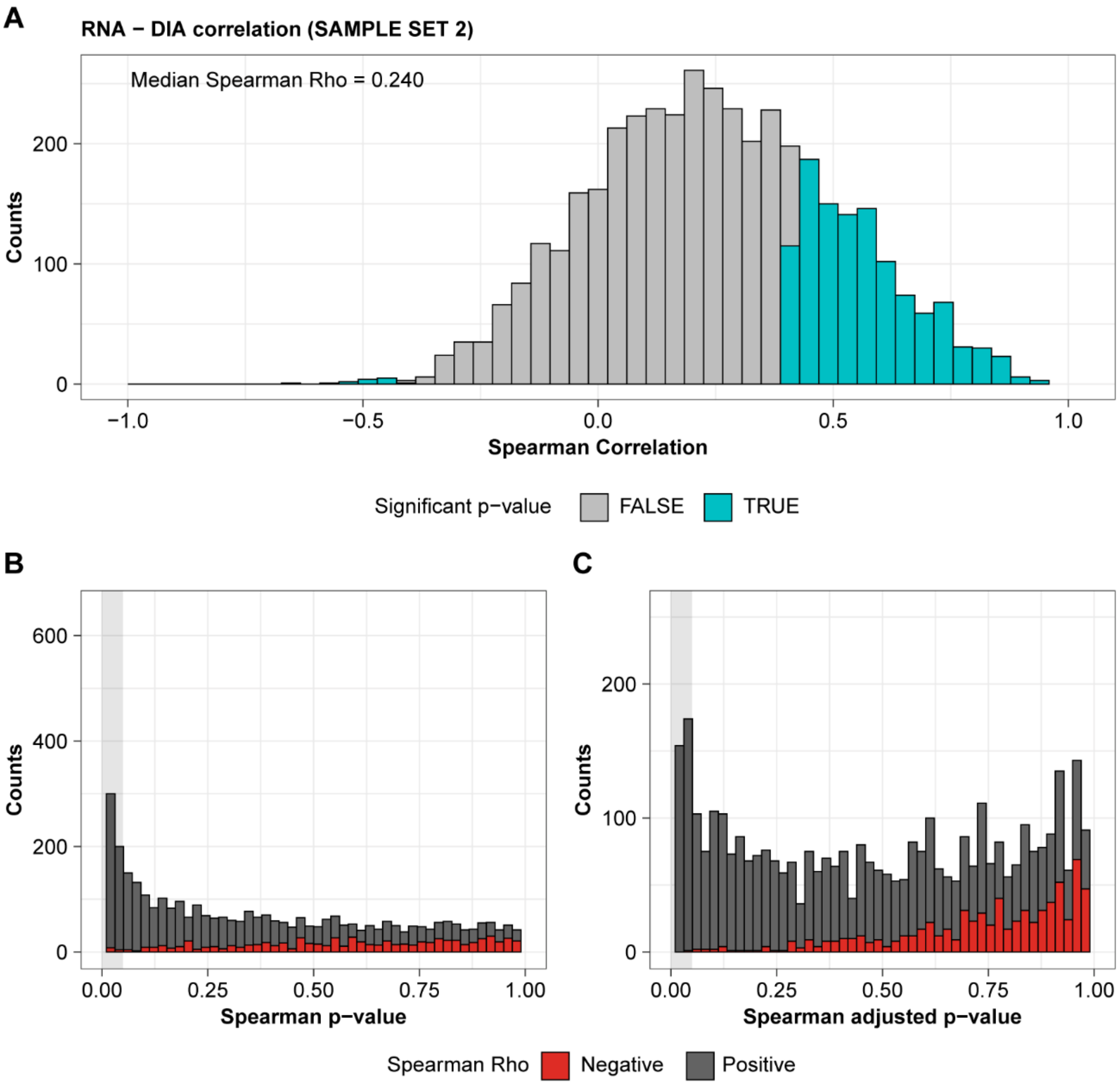

Figure S6. Correlation distribution between RNA and proteomic data in the validation dataset.

Distribution of Spearman Rho correlations of peptides measured by RNA and DIA MS is shown in panel A. Bottom panels display the distribution of p-values (B) and adjusted p-values (C) of positive (dark-gray) and negative (dark red) correlations.

Abbreviations: DIA: Data Independent Acquisition; MS: Mass Spectrometry.

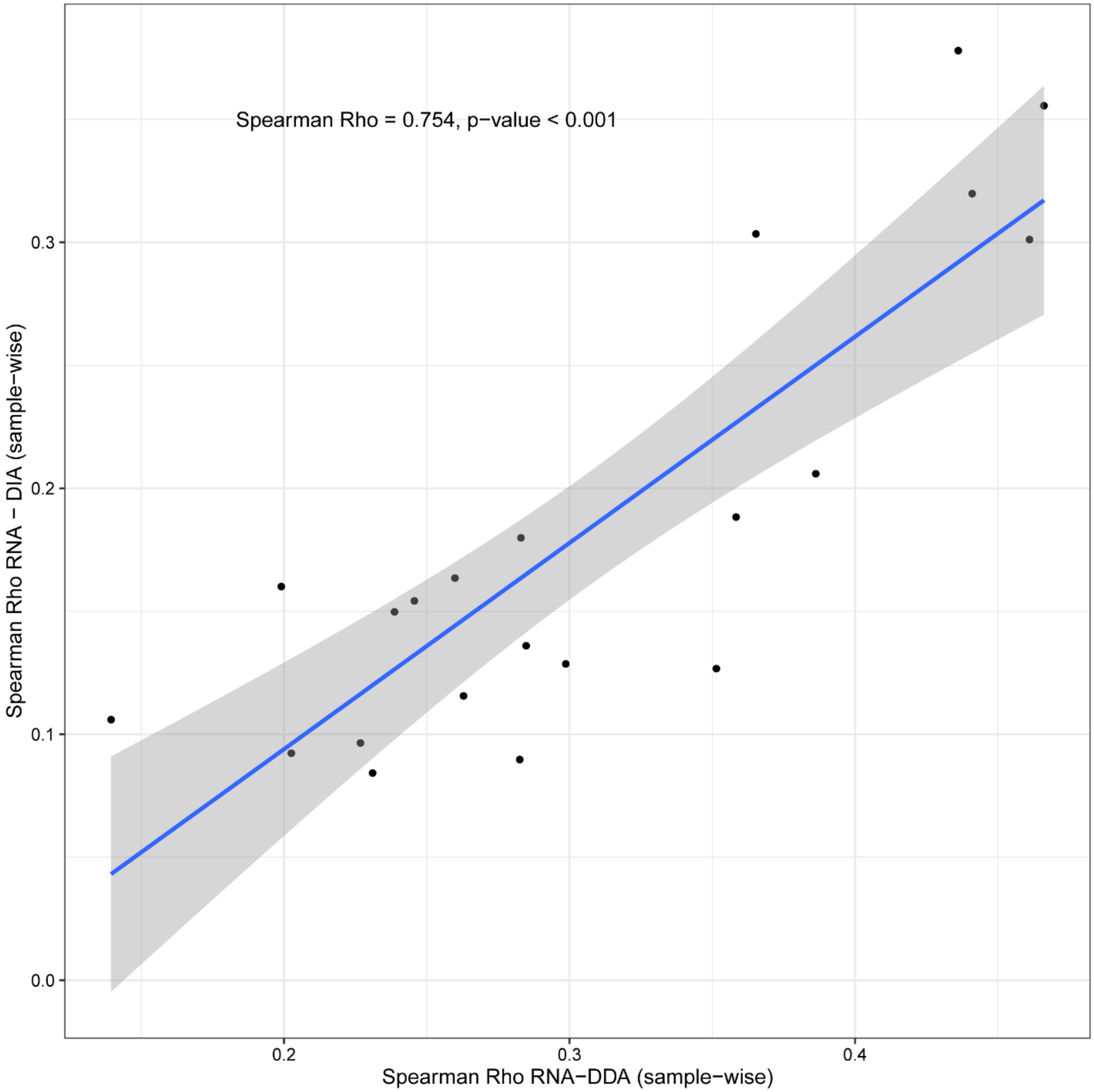

Figure S7. Correlation of RNA-protein correlations between DDA and DIA data layers.

Figure displays correlation between RNA-DDA and RNA-DIA correlations. Linear regression curve and its 95% confidence interval are represented in blue and gray, respectively.

Abbreviations: DDA: Data Dependent Acquisition; DIA: Data Independent Acquisition.

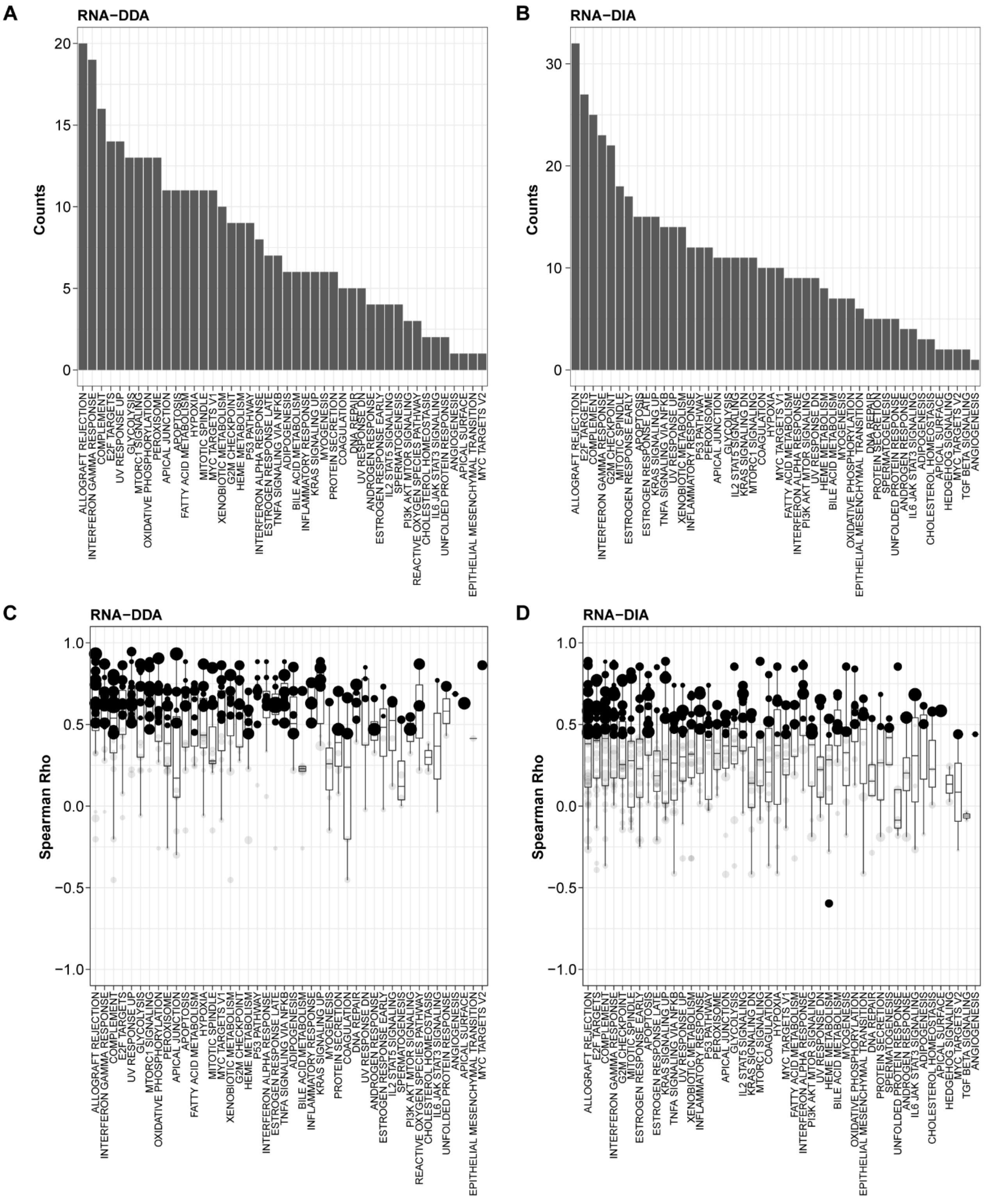

Figure S8. Significant transcript-protein pairs pathway-wise annotation and correlations.

Figure displays HALLMARK pathway annotation of significant transcripts with a detectable protein (DDA and DIA) out of the differential expression analysis of ER status. Panels A and B show the number of significant transcript-

protein pairs in each pathway out of DDA and DIA data, respectively. Panels C and D display RNA-protein correlation distributions in each pathway (gray: non-significant correlation; black: significant correlation) for DDA and DIA data, respectively.

Abbreviations: DDA: Data Dependent Acquisition; DIA: Data Independent Acquisition.

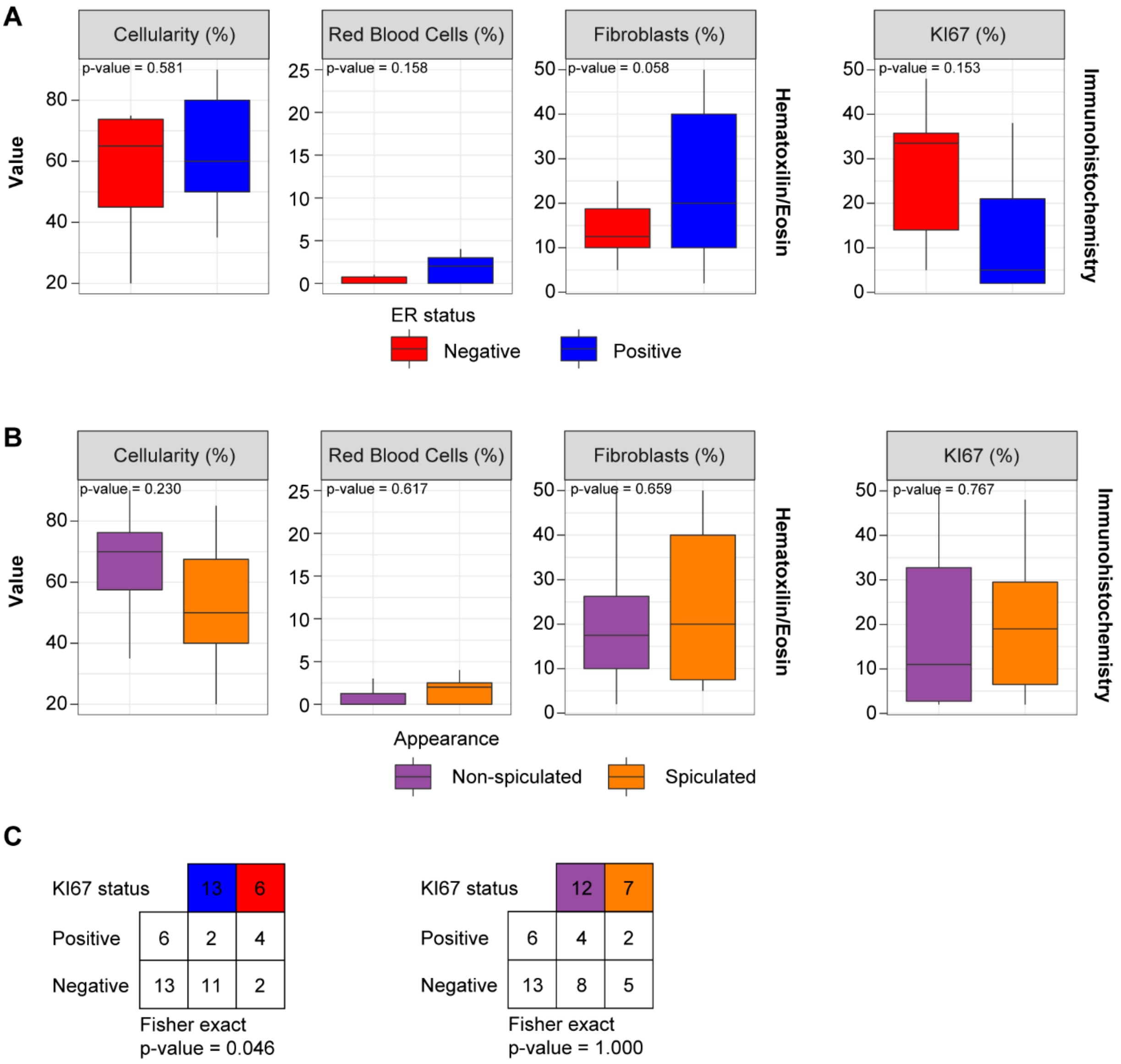

Figure S9. Analysis of immunohistochemical stainings.

Number of tumor cells, red blood cells, and fibroblasts (i.e. hematoxylin-eosin stainings) were analyzed for 19 out of 21 tissues for both ER status and mammographic appearance groups (panel A and B). Quantitative immunohistochemical scores (KI67: percentage of positive tumor cells) were also evaluated (panel A and B, left). Results for KI67 clinical cutoff (≥ 30% positive cells) are depicted as contingency tables in panel C.

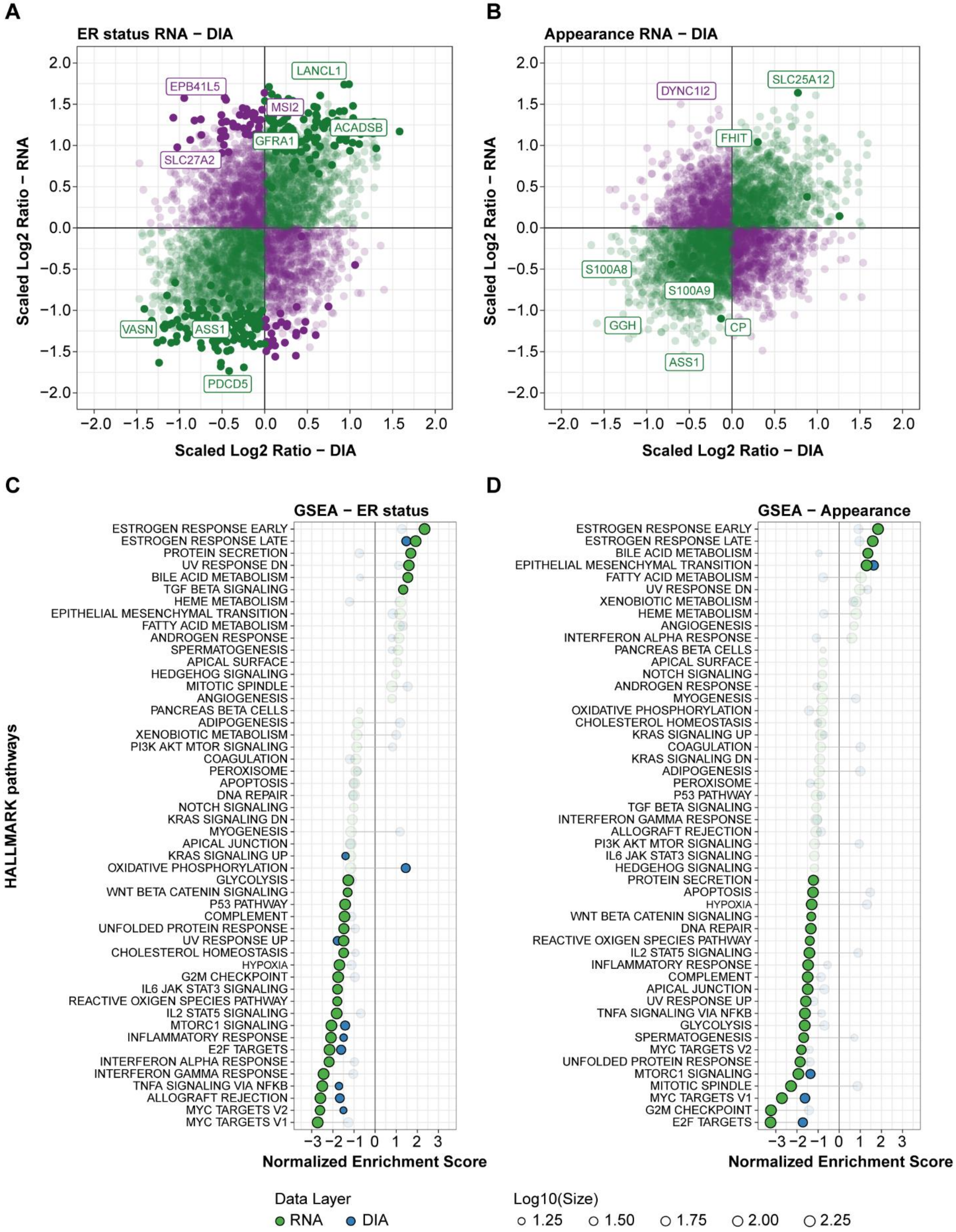

A
ER status RNA − DIA
EPB41L5
LANCL1
MSI2
ACADSB
GFRA1
SLC27A2
VASN
ASS1
PDCD5
Scaled Log2 Ratio − RNA
Scaled Log2 Ratio − DIA
B
Appearance RNA − DIA
DYNC1I2
SLC25A12
FHIT
S100A8
S100A9
GGH
CP
ASS1
Scaled Log2 Ratio − RNA
Scaled Log2 Ratio − DIA
C
GSEA − ER status
ESTROGEN RESPONSE EARLY
ESTROGEN RESPONSE LATE
PROTEIN SECRETION
UV RESPONSE DN
BILE ACID METABOLISM
TGF BETA SIGNALING
HEME METABOLISM
EPITHELIAL MESENCHYMAL TRANSITION
FATTY ACID METABOLISM
ANDROGEN RESPONSE
SPERMATOGENESIS
APICAL SURFACE
HEDGEHOG SIGNALING
MITOTIC SPINDLE
ANGIOGENESIS
PANCREAS BETA CELLS
ADIPOGENESIS
XENOBIOTIC METABOLISM
PI3K AKT MTOR SIGNALING
COAGULATION
PEROXISOME
APOPTOSIS
DNA REPAIR
NOTCH SIGNALING
KRAS SIGNALING DN
MYOGENESIS
APICAL JUNCTION
KRAS SIGNALING UP
OXIDATIVE PHOSPHORYLATION
GLYCOLYSIS
WNT BETA CATENIN SIGNALING
P53 PATHWAY
COMPLEMENT
UNFOLDED PROTEIN RESPONSE
UV RESPONSE UP
CHOLESTEROL HOMEOSTASIS
HYPOXIA
G2M CHECKPOINT
IL6 JAK STAT3 SIGNALING
REACTIVE OXIGEN SPECIES PATHWAY
IL2 STAT5 SIGNALING
MTORC1 SIGNALING
INFLAMMATORY RESPONSE
E2F TARGETS
INTERFERON ALPHA RESPONSE
INTERFERON GAMMA RESPONSE
TNFA SIGNALING VIA NFKB
ALLOGRAFT REJECTION
MYC TARGETS V2
MYC TARGETS V1
HALLMARK pathways
Normalized Enrichment Score
D
GSEA − Appearance
ESTROGEN RESPONSE EARLY
ESTROGEN RESPONSE LATE
BILE ACID METABOLISM
EPITHELIAL MESENCHYMAL TRANSITION
FATTY ACID METABOLISM
UV RESPONSE DN
XENOBIOTIC METABOLISM
HEME METABOLISM
ANGIOGENESIS
INTERFERON ALPHA RESPONSE
PANCREAS BETA CELLS
APICAL SURFACE
NOTCH SIGNALING
ANDROGEN RESPONSE
MYOGENESIS
OXIDATIVE PHOSPHORYLATION
CHOLESTEROL HOMEOSTASIS
KRAS SIGNALING UP
COAGULATION
KRAS SIGNALING DN
ADIPOGENESIS
PEROXISOME
P53 PATHWAY
TGF BETA SIGNALING
INTERFERON GAMMA RESPONSE
ALLOGRAFT REJECTION
PI3K AKT MTOR SIGNALING
IL6 JAK STAT3 SIGNALING
HEDGEHOG SIGNALING
PROTEIN SECRETION
APOPTOSIS
HYPOXIA
WNT BETA CATENIN SIGNALING
DNA REPAIR
REACTIVE OXIGEN SPECIES PATHWAY
IL2 STAT5 SIGNALING
INFLAMMATORY RESPONSE
COMPLEMENT
APICAL JUNCTION
UV RESPONSE UP
TNFA SIGNALING VIA NFKB
GLYCOLYSIS
SPERMATOGENESIS
MYC TARGETS V2
UNFOLDED PROTEIN RESPONSE
MTORC1 SIGNALING
MITOTIC SPINDLE
MYC TARGETS V1
G2M CHECKPOINT
E2F TARGETS
Normalized Enrichment Score
Data Layer
RNA
DIA
Log10(Size)
1.25
1.50
1.75
2.00
2.25

Figure S10. Confirmation of integrated findings in the validation dataset.

Panels A and B display all transcript-protein pairs scaled Log2Ratios for ER status and appearance, respectively. Significant differential expression at the RNA level is marked by full dots and in bigger size; concordance and discordance between RNA and protein layers are shown in green and purple, respectively). GSEA analyses were performed on RNA and DIA data layers for ER and spiculation statuses using the Hallmark database. Pathways are ranked based on RNA-level enrichment score. Panel C displays overlap of GSEA analyses for ER status, while panel D shows results of analysis of appearance features (i.e. spiculation vs no-spiculation). Significant pathways in each data layer (RNA: green; DIA: blue) are marked in full color, while transparent ones did not pass the False Discovery Rate (FDR < 0.25) cutoff. Positive scores mark enrichment in ER positive and spiculated tumors, respectively, while negative scores define enrichments in ER negative and non-spiculated samples.

Acronyms: DIA: Data Independent Acquisition; ER: Estrogen Receptor; FDR: False Discovery Rate; GSEA: Gene Set Enrichment Analysis.

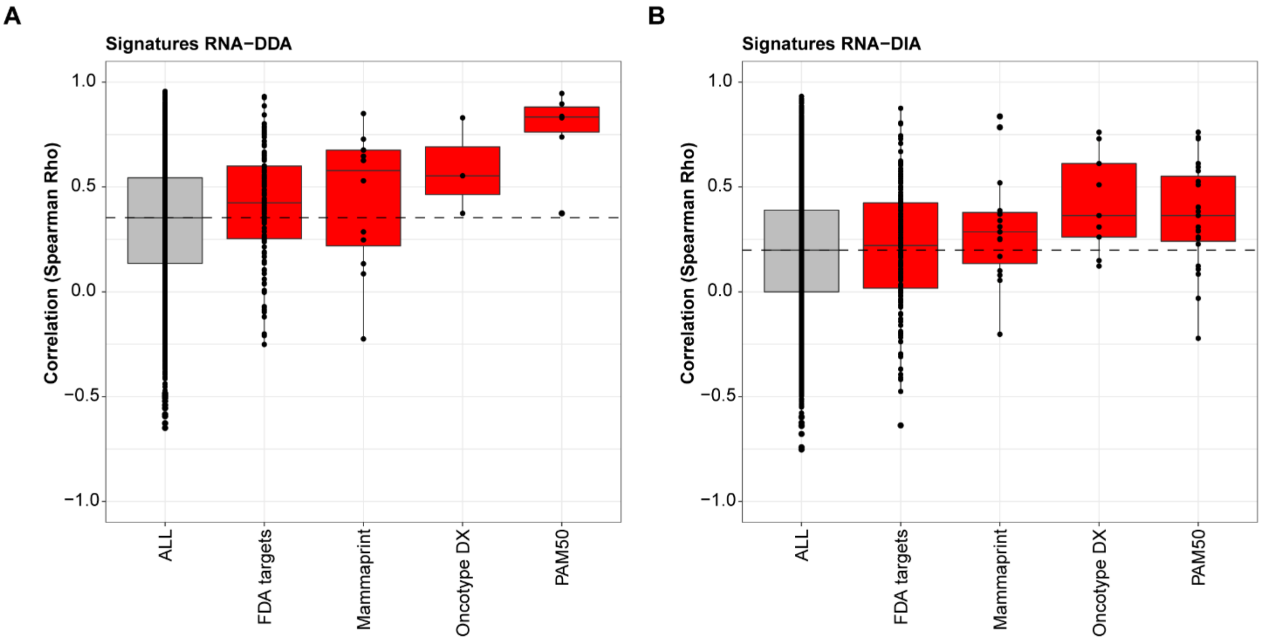

Figure S11. Evaluation of biomarker signatures.

Figure show correlation between transcript-protein pairs for biomarker signatures (Mammaprint, Oncotype-DX, and the PAM50 classifier) for both the DDA (A) and DIA (B) data layers.

Abbreviations: DDA: Data Dependent Acquisition; DIA: Data Independent Acquisition; ER: Estrogen Receptor; FDA: Food and Drug Administration.

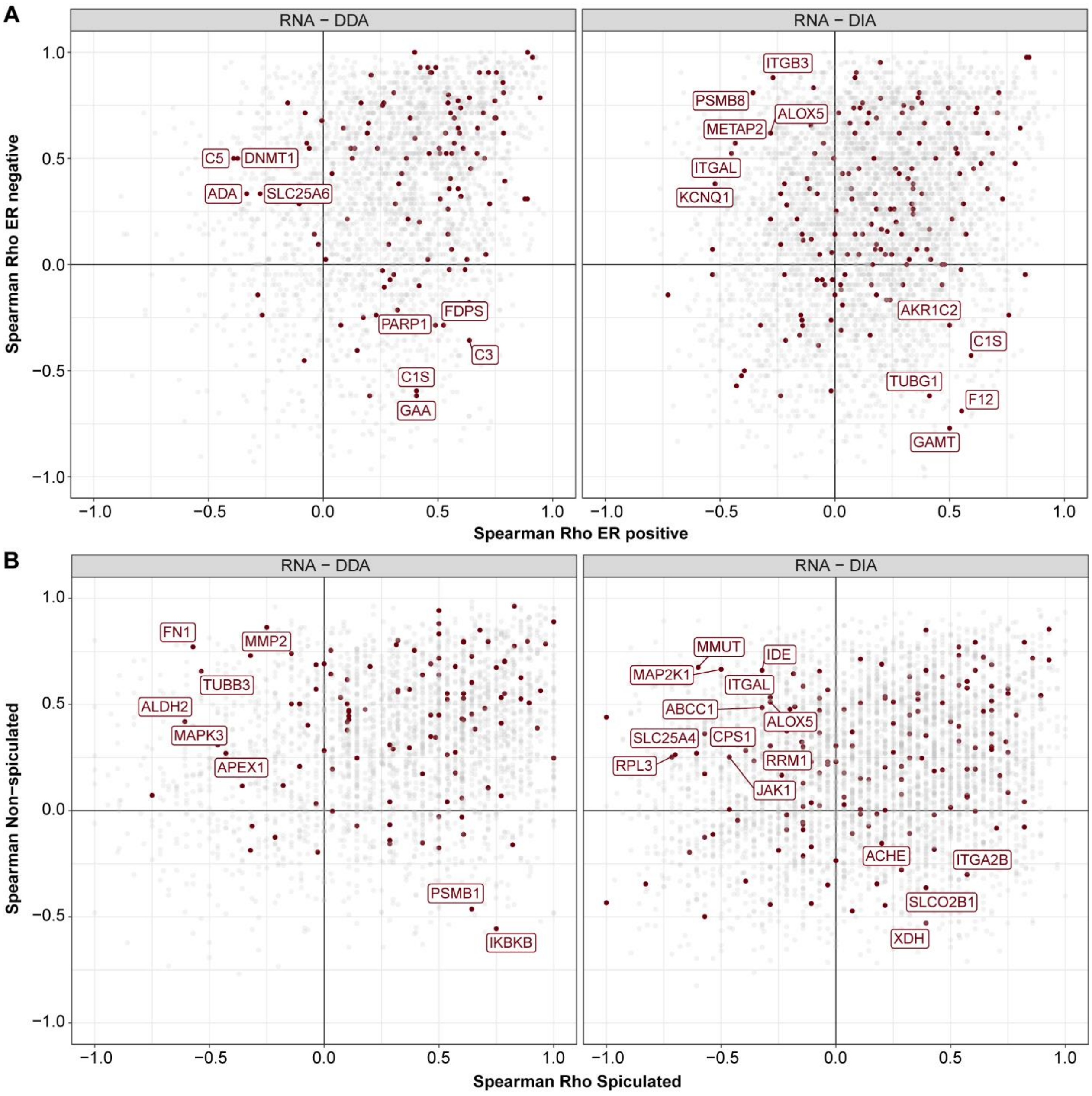

Figure S12. RNA-protein correlations based on tumor subgroup.

Figure displays overall RNA-protein correlations within tumor subgroups (panel A: ER status; panel B: appearance). Left and right panels show comparison with DDA and DIA data, respectively. Proteins marked in dark red represent FDA drug targets.

Abbreviations: DDA: Data Dependent Acquisition; DIA: Data Independent Acquisition; ER: Estrogen Receptor; FDA: Food and Drug Administration.

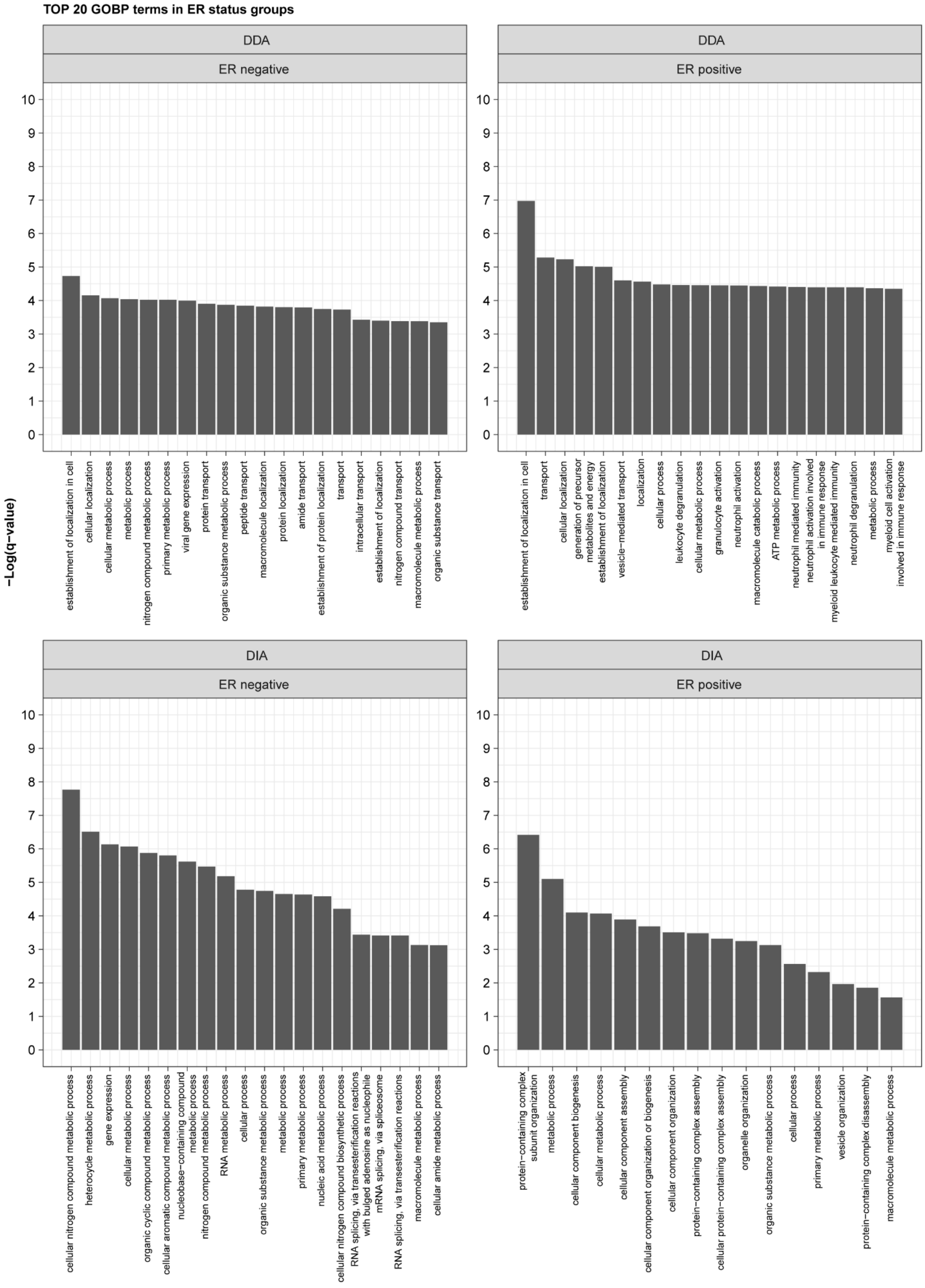
TOP 20 GOBP terms in ER status groups
−Log(q−value)
DDA
ER negative
establishment of localization in cell
cellular localization
cellular metabolic process
metabolic process
nitrogen compound metabolic process
primary metabolic process
viral gene expression
protein transport
organic substance metabolic process
peptide transport
macromolecule localization
protein localization
amide transport
establishment of protein localization
transport
intracellular transport
establishment of localization
nitrogen compound transport
macromolecule metabolic process
organic substance transport
DDA
ER positive
establishment of localization in cell
transport
cellular localization
generation of precursor metabolites and energy
establishment of localization
vesicle−mediated transport
localization
cellular process
leukocyte degranulation
cellular metabolic process
granulocyte activation
neutrophil activation
macromolecule catabolic process
ATP metabolic process
neutrophil mediated immunity
neutrophil activation involved in immune response
myeloid leukocyte mediated immunity
neutrophil degranulation
metabolic process
myeloid cell activation involved in immune response
DIA
ER negative
cellular nitrogen compound metabolic process
heterocycle metabolic process
gene expression
cellular metabolic process
organic cyclic compound metabolic process
cellular aromatic compound metabolic process
nucleobase−containing compound metabolic process
nitrogen compound metabolic process
RNA metabolic process
cellular process
organic substance metabolic process
metabolic process
primary metabolic process
nucleic acid metabolic process
cellular nitrogen compound biosynthetic process
RNA splicing, via transesterification reactions with bulged adenosine as nucleophile
mRNA splicing, via spliceosome
RNA splicing, via transesterification reactions
macromolecule metabolic process
cellular amide metabolic process
DIA
ER positive
protein−containing complex subunit organization
metabolic process
cellular component biogenesis
cellular metabolic process
cellular component assembly
cellular component organization or biogenesis
cellular component organization
protein−containing complex assembly
cellular protein−containing complex assembly
organelle organization
organic substance metabolic process
cellular process
primary metabolic process
vesicle organization
protein−containing complex disassembly
macromolecule metabolic process
0
1
2
3
4
5
6
7
8
9
10

Figure S13. Pathway representation of transcript-protein pairs displaying discordant correlations between ER positive and negative tumors.

Figure displays the top 20 significant GOBP pathways derived from the Panther over-representation test for transcript-protein pairs between ER positive and negative groups. Top panels display results for the DDA data, while DIA results are shown in the bottom panels (left panels: ER negative; right panels: ER positive).

Abbreviations: DDA: Data Dependent Acquisition; DIA: Data Independent Acquisition; ER: Estrogen Receptor; GOBP: Gene Ontology Biological Process.

TOP 20 GOBP terms in Appearance groups

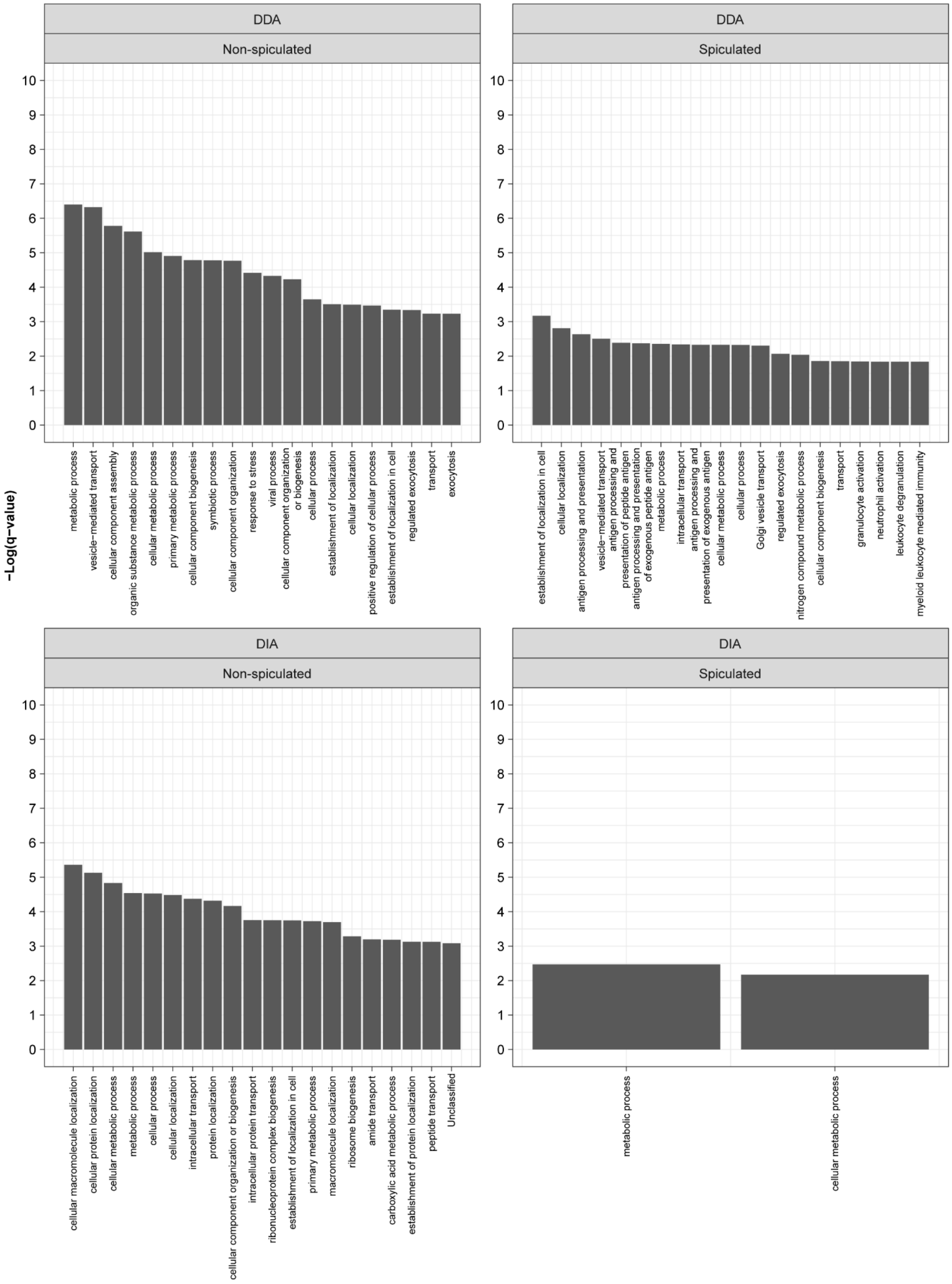

Figure S14. Pathway representation of transcript-protein pairs displaying discordant correlations between tumor appearance groups.

Figure displays the top 20 significant GOBP pathways derived from the Panther over-representation test for transcript-protein pairs between spiculated and non-spiculated tumors. Top panels display results for the DDA data, while DIA results are shown in the bottom panels (left panels: spiculated tumors; right panels: non-spiculated tumors).

Abbreviations: DDA: Data Dependent Acquisition; DIA: Data Independent Acquisition; GOBP: Gene Ontology Biological Process.

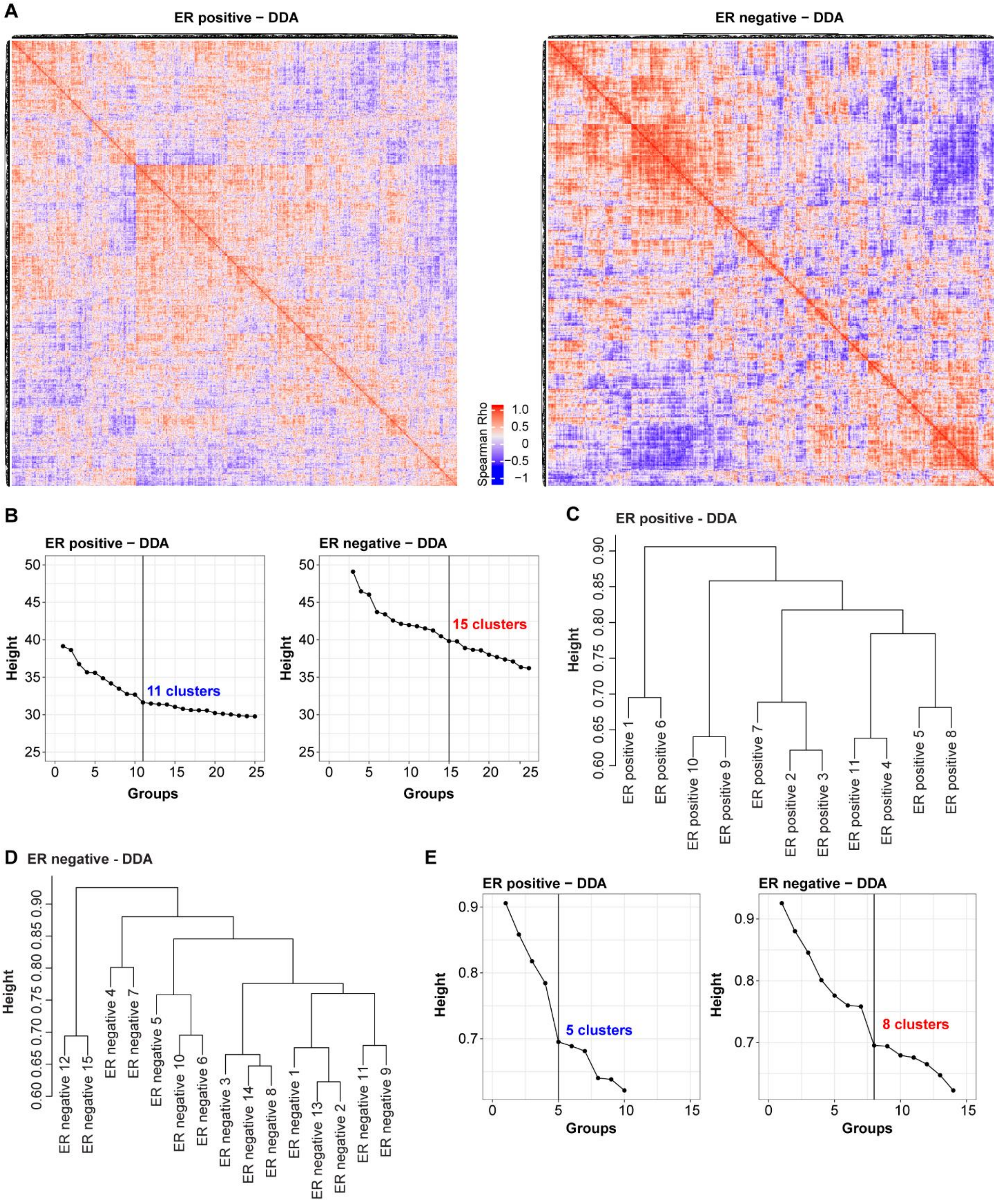

Figure S15. Co-regulation clusters in ER positive and negative tumor samples out of the DDA data layer. In order to define the most represented pathways in our DDA MS data, we generated Spearman correlation matrices within the ER positive and ER negative tumor groups (A). The elbow method was used to define the minimum number

of clusters for both sample groups (ER positive: 11 clusters; ER negative: 15 clusters; panel B). Highly similar clusters based on Panther over-representation test-derived GOBP annotation were merged after calculating the distances between them in the ER positive (C) and negative (D) subgroups, and by selecting a new minimum number of protein clusters (E).

Abbreviations: DDA: Data Dependent Acquisition; ER: Estrogen Receptor; GOBP: Gene Ontology Biological Process.

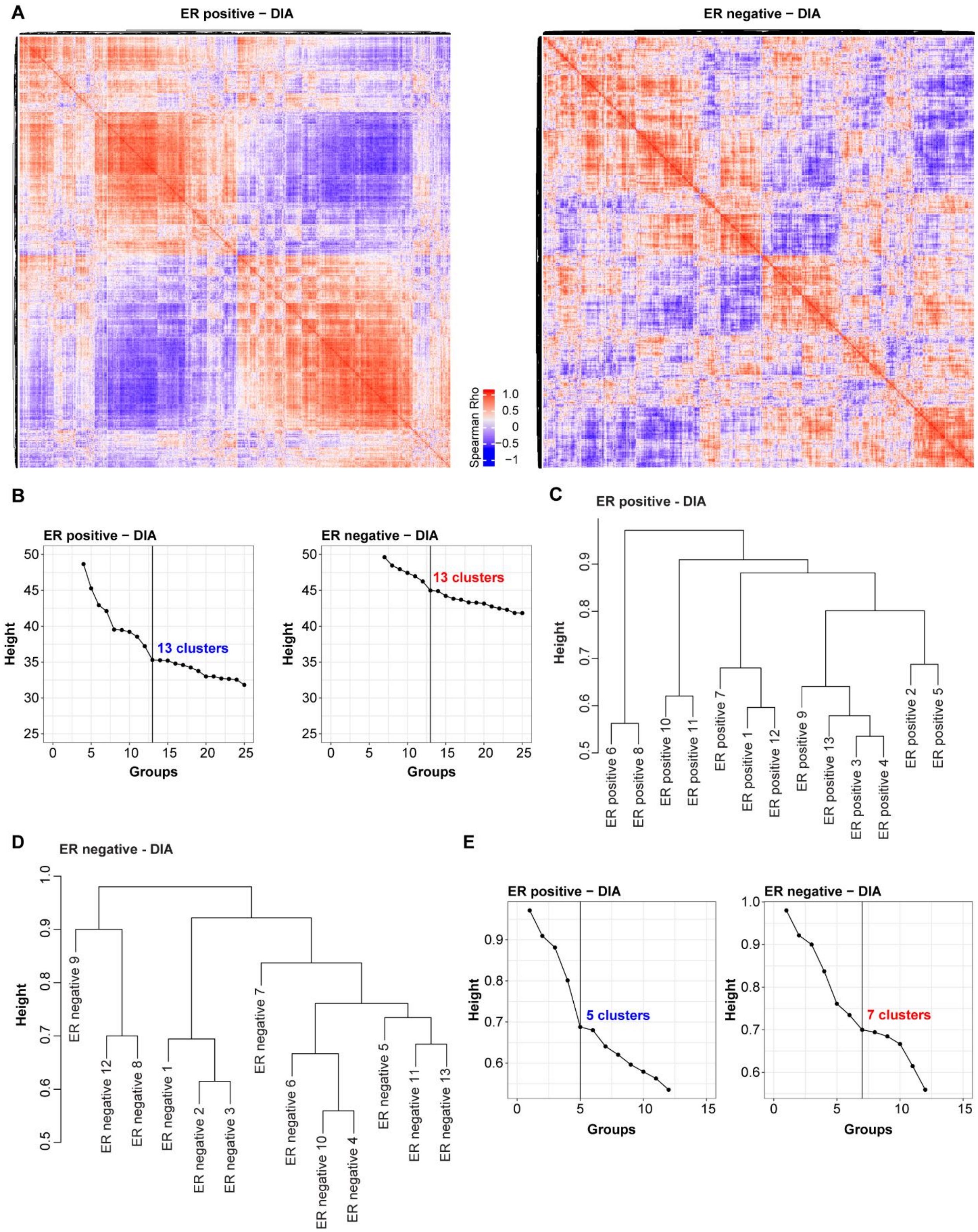

Figure S16. Co-regulation clusters in ER positive and negative tumor samples out of the DIA data layer.

Following our analysis of clusters for DIA data, we generate Spearman correlation matrices within the ER positive and ER negative tumor groups (A) out of the DIA data layer. The elbow method was used to define the minimum number of clusters for both sample groups (ER positive: 13 clusters; ER negative: 13 clusters; panel B). Highly similar clusters based on Panther over-representation test-derived GOBP annotation were merged after calculating the distances between them in the ER positive (C) and negative (D) subgroups, and by selecting a new minimum number of protein clusters (E).

Abbreviations: DIA: Data Independent Acquisition; ER: Estrogen Receptor; GOBP: Gene Ontology Biological Process.

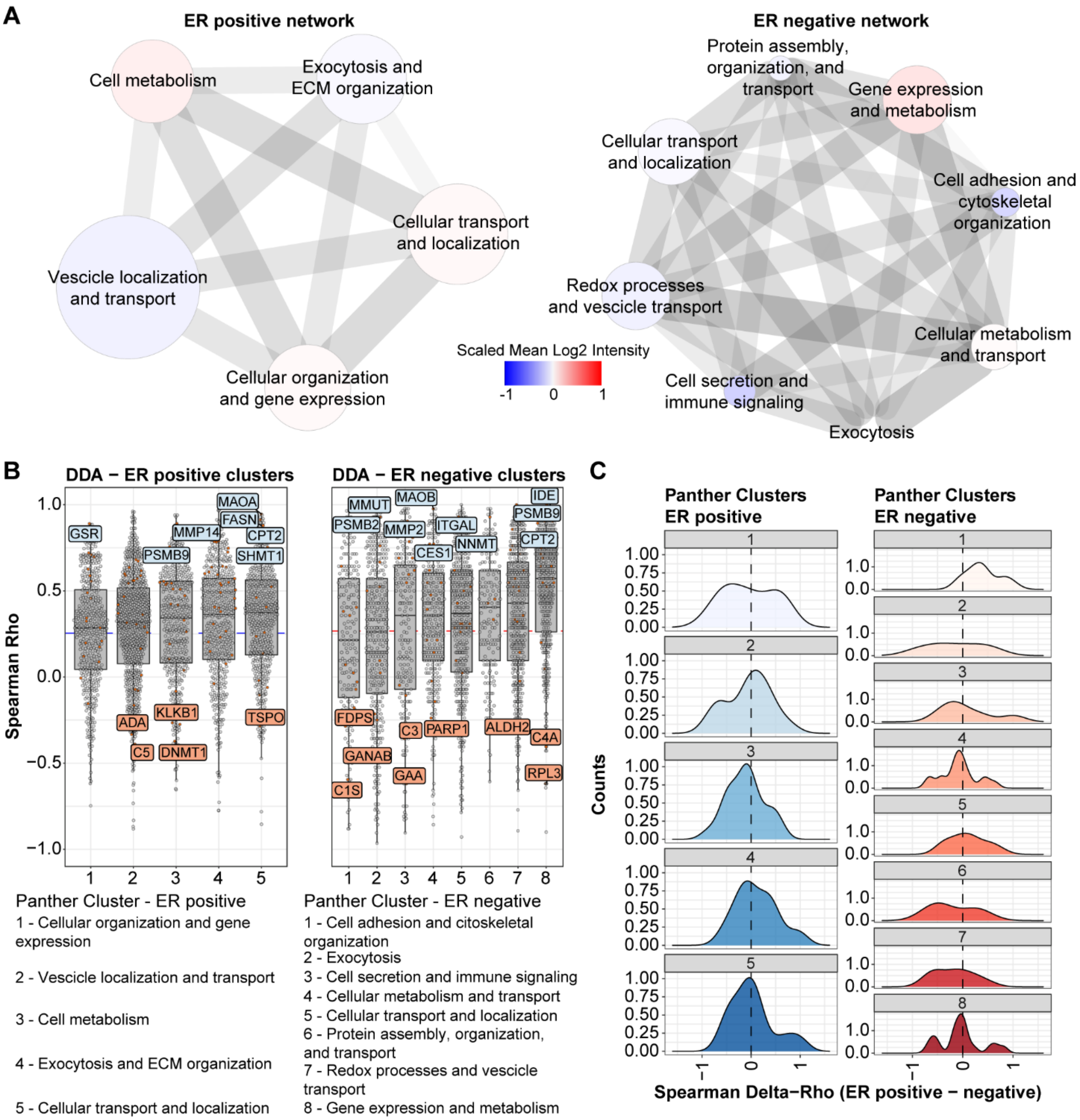

Figure S17. Protein cluster regulation dependent on Estrogen Receptor status out of the DDA data layer.

Co-regulated protein clusters in ER positive (left) and negative (right) tumors (see Fig S14) were extracted from the DDA data, annotated with GOBP terms, condensed, and visualized in Cytoscape (panel A). Edge thickness and length relates to cluster distance (Euclidean), node color relates to the scaled mean intensity of all protein in each cluster, and node size depends on the number of proteins in each cluster.

Panel B shows the correlation to mRNA of each protein per cluster for ER positive and negative tumors. Panel C displays the distributions of the differences between Spearman correlation coefficients between ER positive and negative tumors across clusters of co-regulation (FDA drug targets only).

Abbreviations: DDA: Data Dependent Acquisition ER: Estrogen Receptor; FDA: Food and Drug Administration.

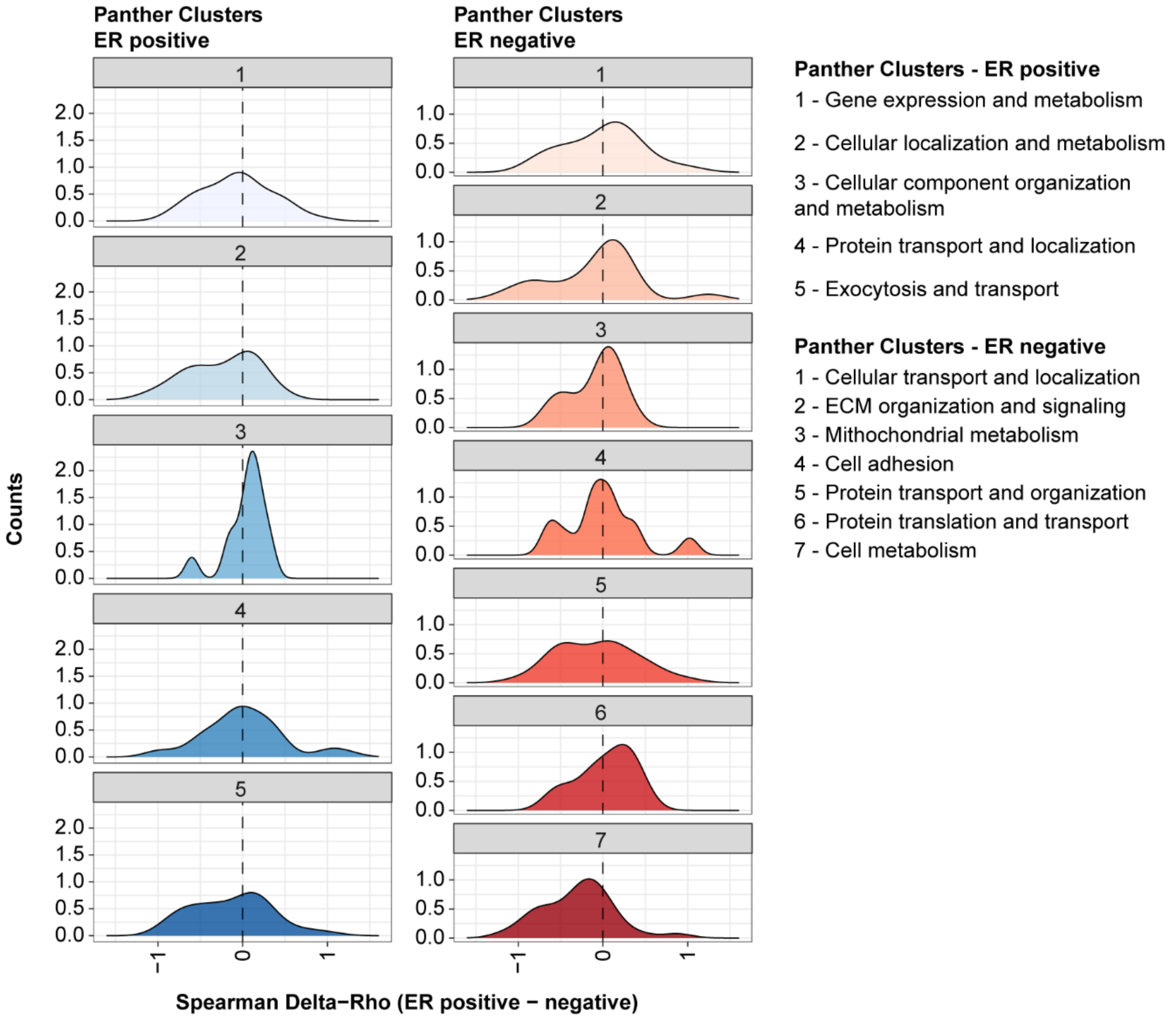

Figure S18. Difference in correlation coefficients in clusters of regulation dependent on Estrogen Receptor status out of the DIA data layer.

Figure displays distributions of the differences between Spearman correlation coefficients between ER positive and negative tumors across clusters of co-regulation (FDA drug targets only).

Acronyms: DIA: Data Independent Acquisition; FDA: Food and Drug Administration.
